# Coupled CFD–DEM analysis of soil cavity formation and gas diffusion during buried pipeline leakage

**DOI:** 10.1098/rsos.260883

**Published:** 2026-10-14

**Authors:** Fanxi Bu, Qingxiu Lu, Yuheng He, Bohan Jia, Zhuoran Lv, Zhihua Wang

**Affiliations:** ^1^ Key Laboratory for Enhanced Oil & Gas Recovery of the Ministry of Education, Northeast Petroleum University, Daqing, People's Republic of China; ^2^ School of English and International Studies, Beijing Language and Culture University, Beijing, People's Republic of China

**Keywords:** buried pipelines, gas leakage diffusion, soil structure deformation, particle motion, CFD–DEM coupling

## Abstract

After the leakage of a buried pipeline, the overlying soil structure deforms under the high-speed impact of escaping gas, influencing both leakage and diffusion behaviours. This deformation typically appears as a soil cavity above the leakage port, especially in medium- and low-pressure conditions. In this study, the formation process of soil cavities is investigated using CFD–DEM coupled numerical simulations. The control variable method is applied to examine how different conditions affect cavity size and gas diffusion, and the sensitivity between cavity characteristics and leakage behaviour is analysed. Results show that the leaked gas forms a high-velocity zone near the leakage port, generating negative pressure in the surrounding area. Soil cavities generally form and stabilize within 0.1 s under gas impact. Soil particle size and porosity significantly influence the cavity formation time by altering the pore structure and total pore volume. The average sensitivity coefficients of the maximum cavity width to gas leakage velocity and the highest dangerous range (HDR) are 5.71 and 18.78, respectively, indicating that larger cavity widths correspond to higher leakage velocities and greater HDR. Therefore, the maximum cavity width serves as the most appropriate indicator for evaluating soil cavities caused by buried gas pipeline leakage.

## 1. Introduction

As a form of clean energy with high calorific value and abundant reserves, natural gas is regarded as an important energy source in the transition from the global energy structure to renewable energy because the carbon emissions and pollutants caused by combustion are far less than those of traditional fossil energy, such as oil and coal [1,2]. In recent years, global natural gas consumption has been rising, and in 2023, global natural gas consumption reached 4.01 trillion cubic metres, accounting for more than 20% of the global energy supply structure [3,4].

The huge consumer demand has put forward higher requirements for the transportation and storage of natural gas. The transportation status of natural gas is mainly divided into gaseous natural gas and LNG. LNG is often transported by waterway and road, while most gaseous natural gas is transported by pipeline [5]. Pipeline transportation of natural gas has a series of advantages, such as large volume, high efficiency and low cost, and the incidence of pipeline transportation accidents is much lower than that of road transportation. However, owing to the high population density and commercial construction density in the gas pipeline laying area, the casualties and economic losses caused by leakage accidents are much higher than those caused by road transportation [6]. Gas pipelines are often laid underground. Owing to the concealed and complex buried environment of the pipelines, as well as the construction of urban underground spaces being complex and ever-changing, leakage accidents occur from time to time owing to corrosion, natural disasters and third-party damage. As the most common accident of pipelines, leakage will not only cause damage to public facilities, economic losses and environmental pollution, but also cause secondary explosion accidents [7].

Owing to the resistance of the buried soil above a pipeline, the leakage of buried pipelines is significantly different from that of other pipelines. Many scholars have conducted extensive research on the gas diffusion after the leakage of buried gas pipelines. Cho *et al.* [8] collected on-site data from 77 accidents and analysed the impact of different soil characteristics on methane leakage and diffusion through on-site soil sampling. The study showed that high soil moisture content can increase the horizontal diffusion distance of methane in soil. Latifi *et al.* [9] conducted indoor experiments to investigate the impact of various soil properties on the leakage from polyethylene pipe cracks. The findings revealed that soil particle size, permeability coefficient and dry density significantly influenced the leakage volume. Based on the experimental data, quantitative relationships were established between characteristic soil parameters, such as permeability coefficient, porosity and dry unit weight, and the FAVAD model coefficients *C_d_* and *m*, respectively. Yu *et al.* [10] conducted a large-scale experiment on small-hole leakage in buried CO_2_ pipelines, analysing the leakage patterns and temperature evolution in the soil area under varying leakage aperture sizes and directions. The study revealed a significant difference between buried pipeline leakage and above ground pipeline leakage. The soil obstructs the gas jet, causing dry ice pellets to accumulate in soil and preferentially spread to areas with lower soil resistance. Ulrich *et al.* [11], taking into account the type of backfill soil, surface wind speed, atmospheric environment and other on-site factors, conducted experimental measurements of the concentration distribution of gas leakage from buried gas pipelines into the atmosphere; it was found that the wet soil cover would hinder the diffusion of gas into the atmosphere above the soil. Liu *et al.* [12] established a shallow sand low-pressure buried pipeline leakage experiment, revised the prediction model of the buried pipeline leakage rate through experiments and numerical simulations, analysed the factors affecting the pipeline leakage rate and concluded that the soil filling depth had little influence on the buried pipeline leakage rate, while the soil particle diameter and porosity had a greater influence. The study by Mohanty *et al.* [13] based on Liu further improved the distribution range of combustible clouds in which methane leaks into the air after the leakage of buried gas pipelines. Bagheri *et al.* [14] compared the leakage situation of buried gas pipelines and above ground gas pipelines and found that the resistance effect of soil has a significant impact on the diffusion speed of leaked gas, reducing its velocity from supersonic flow to subsonic flow. At the same time, the influence of pipe wall thickness on underground gas pipeline leakage is analysed. It was found that considering pipe wall thickness will obviously changes the soil temperature field near the pipeline, and a prediction model of leakage rate was fitted according to the simulation results. Zheng *et al.* [15] carried out numerical simulations on the leakage of buried gas pipelines under various soil layered backfill conditions. The findings revealed that layered backfill alters the distinctly layered distribution of gas concentration. Under different backfill conditions, the risk levels following gas leakage also vary. Specifically, when the first layer above the pipeline is back-filled with clay, the diffusion range of the leaked gas is effectively minimized, and its associated risk level is notably lower than that of other backfill methods. Bezaatpour *et al.* [16] established a three-dimensional anisotropic layered soil model based on real soil characteristic data collected from different regions and assumed that the transported gas was a multi-component gas, making the leaked gas closer to the real leakage state. The study showed that the gas diffusion results in anisotropic soil were significantly different from those in previous isotropic soil; the concentration profile showed an uneven diffusion state, and the changes in the mechanical and hydraulic properties of each layer of soil had a significant impact on gas diffusion.

A large number of related studies on the leakage of buried gas pipelines show that the characteristics of soil overlying pipelines are one of the important factors affecting gas leakage and diffusion [17,18]. With the continuous deepening of research, relevant studies have shown that the leakage and diffusion of buried gas pipelines can change the soil's own structure, thereby affecting gas leakage and diffusion. Houssin-Agbomson *et al.* [19] conducted full-scale experiments on leakage of high-pressure buried pipelines. The experimental results indicate that the soil structure changes caused by high-pressure pipeline leaks can affect the soil surface layer. At 7.8 MPa pipeline operating pressure, the soil surface forms an obvious crater under the impact of leaked gas. At this time, the diffusion of leaking gas can be considered as a free jet. Even when the operating pressure of the pipeline decreases to 1.7 MPa, changes in the surface structure of the soil can still be observed in loose sandy soils. Bonnaud *et al.* [20] divided the changes in soil structure caused by leaking gas into migration and cratering. When the pressure of the pipeline is higher and the depth of the pipeline is smaller, the probability of the leakage gas impacting the surface of the soil to form a crater is greater. At the same time, taking soil moisture content as the experimental variable, it is found that when the soil moisture content decreases, the internal cohesion of the soil will be reduced, resulting in an increased probability of surface impact crater formation. Wang *et al.* [21] studied the internal flow field of soil after the leakage of a sub-high-pressure buried gas pipeline using the CFD–DEM coupling method. Owing to the appearance of a high-speed jet in the cavity area at the leakage port, the peak velocity reaches 1000 m s^−1^, and the sound field imaging using the quadrupole sound source generated by high-speed flow can realize the location of the pipeline leakage point. Zhang *et al.* [22] studied the multi-point source leakage of buried gas pipelines and found that multi-point source leakage is more destructive to the soil surface than single-source leakage and that the soil morphology changes caused by leakage are also different. Single-source leakage and dual-source leakage can cause ‘linear’ damage to the soil surface, and multi-point source leakage can also cause irregular collapse holes. Zhao *et al.* [23] constructed a two-dimensional visual buried gas pipeline leakage experimental box. According to the experimental results, the soil morphology formed by leakage impact was divided into pore zone, main crack diffusion zone and fully developed micro-crack zone. The increase in pipeline pressure promoted the formation of pores, while the increase in soil moisture content inhibited the formation of pores. Li *et al.* [24] employed the CFD–DEM coupling method to investigate soil erosion and surface collapse induced by the leakage of buried water pipes. The findings revealed that following the leakage, soil particles initially migrated towards the leakage orifice, followed by a gradual upward expansion of the cavity, ultimately resulting in surface subsidence or collapse.

In summary, with the deepening of related studies on the leakage of buried gas pipelines, a preliminary understanding of the damage to the soil internal structure caused by leakage gas impact has been obtained. The operating conditions of pipelines and the characteristics of soil itself can affect the formation process and final state of soil cavities, but most studies focus on the structure of soil voids under stable conditions. This is because the formation process of soil voids is located inside the soil, which is difficult to observe experimentally. At the same time, there are few studies on the influence of soil cavities on the leakage and diffusion of buried gas pipelines. The evolution law of soil cavities owing to the leakage of buried medium- and low-pressure gas pipelines is still insufficient, and the influence of cavity formation on the diffusion of gas leakage also lacks systematic research.

Aiming at soil internal deformation behaviour caused by medium-pressure buried gas pipeline leakage, this paper analyses soil internal deformation behaviour and the gas leakage diffusion situation after buried gas pipeline leakage based on CFD–DEM coupling, calculates soil particle movement through DEM and realizes observation of the soil internal deformation process. The controlled variable method is used to study the leakage of buried gas pipelines under different soil particle sizes, porosity, moisture content and pipeline operating pressures. The mutual influence between different soil deformation behaviours and gas leakage diffusion is explored, and an accurate description of gas leakage diffusion behaviour under real soil structure is provided.

## 2. Methods

### 2.1. Physical model

To analyse the soil structure deformation process and gas leakage diffusion law caused by underground gas pipeline leakage, a physical model of the buried gas pipeline, as shown in figure 1a was established. The model considers the influence of pipeline wall thickness; the pipeline diameter is 100 mm, the wall thickness is 5 mm, the burial depth is 600 mm and the leakage port is located directly above the centre of the pipeline with a diameter of 20 mm. To understand the movement of leaked gases inside the soil, corresponding monitoring points and lines were set up in the soil. Figure 1b shows the distribution of monitoring points and monitoring lines in the soil. The direction of monitoring line 1 is vertically upward, extending from the leakage port to the surface of the soil. The specific coordinates of each monitoring point are shown in table 1.

**Figure 1.**
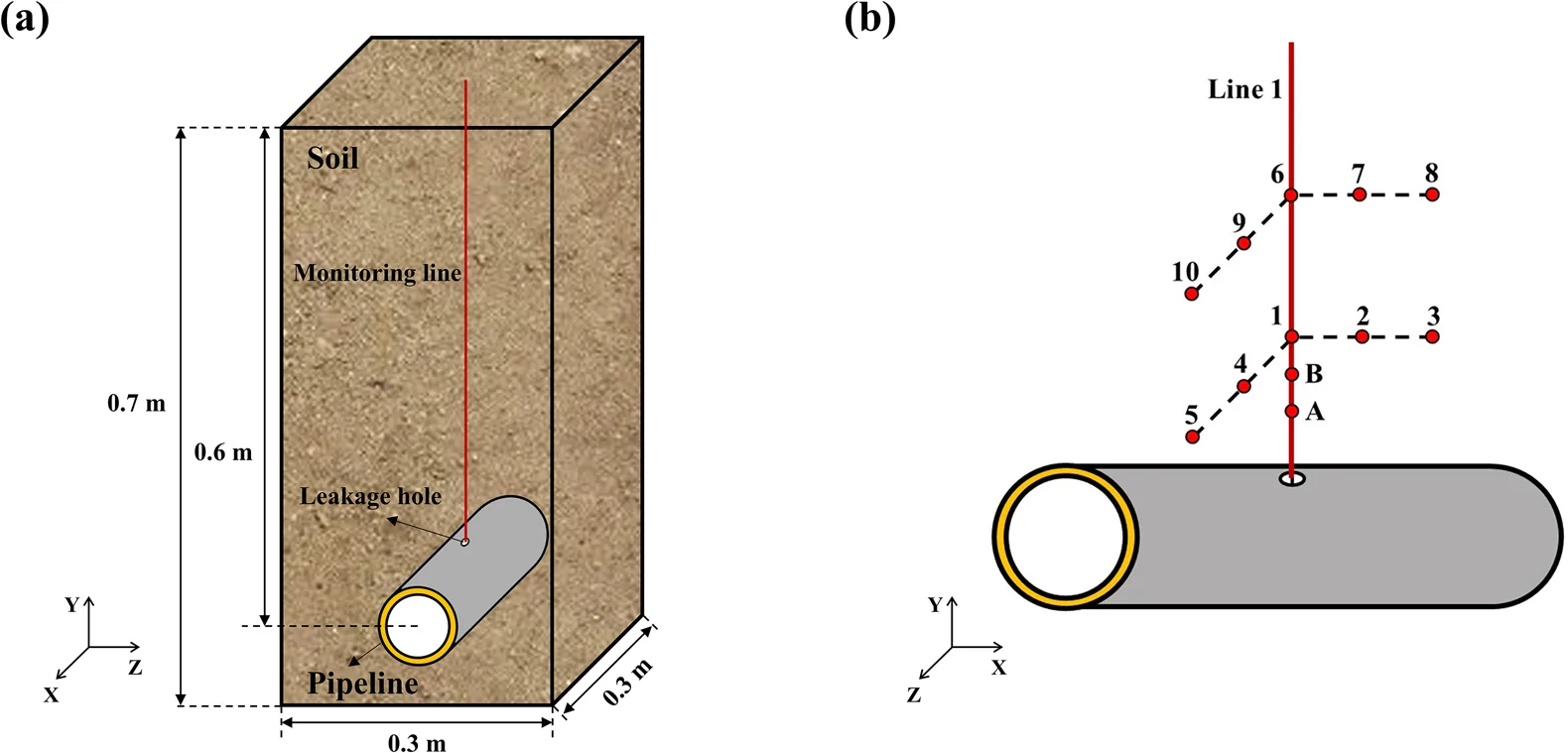
(a) Schematic diagram of physical model and (b) schematic diagram of monitoring points and monitoring line.

**Table 1.** Different coordinates of each monitoring point (coordinate unit mm).

number	position	number	position
point A	(0, 50, 0)	point 5	(0, 100, 100)
point B	(0, 75, 0)	point 6	(0, 200, 0)
point 1	(0, 100, 0)	point 7	(50, 200, 0)
point 2	(50, 100, 0)	point 8	(100, 200, 0)
point 3	(100, 100, 0)	point 9	(0, 200, 50)
point 4	(0, 100, 50)	point 10	(0, 200, 100)

### 2.2. Mathematical modelling

In this paper, the CFD–DEM coupling method is used to simulate the leakage gas movement of a buried gas pipeline and the change of soil internal structure. The soil is idealized as discrete spherical soil particles and the movement results of each particle are calculated to characterize the changes in soil structure. The mathematical model used for simulation consists of three parts: describing the gas motion control equations inside the pipeline and leaking into the soil, describing the particle motion control equations for soil particle collision motion and the CFD–DEM coupling algorithm.

#### 2.2.1. Governing equations used in CFD

The governing equation of gas motion is as follows.

(1) Mass conservation equation [25]:

(2.1) $$\frac{\partial }{\partial t}\left(\varphi_{g}\rho_{g}\right)+\nabla \times \left(\varphi_{g}\rho_{g}u_{g}\right)=0,$$

where *φ*_*g*_ is the volume fraction of the gas in the fluid unit [26], *ρ*_*g*_ is the gas density and *u_g_* is the gas velocity vector.

The ideal gas state equation is used to describe the gas density:

(2.2) $$P=\rho_{g}RT,$$

where *R* is the gas constant, J kg^−1^ K^−1^; *T* is the gas temperature, K and *P* is the pressure, Pa.

**Figure 2.**
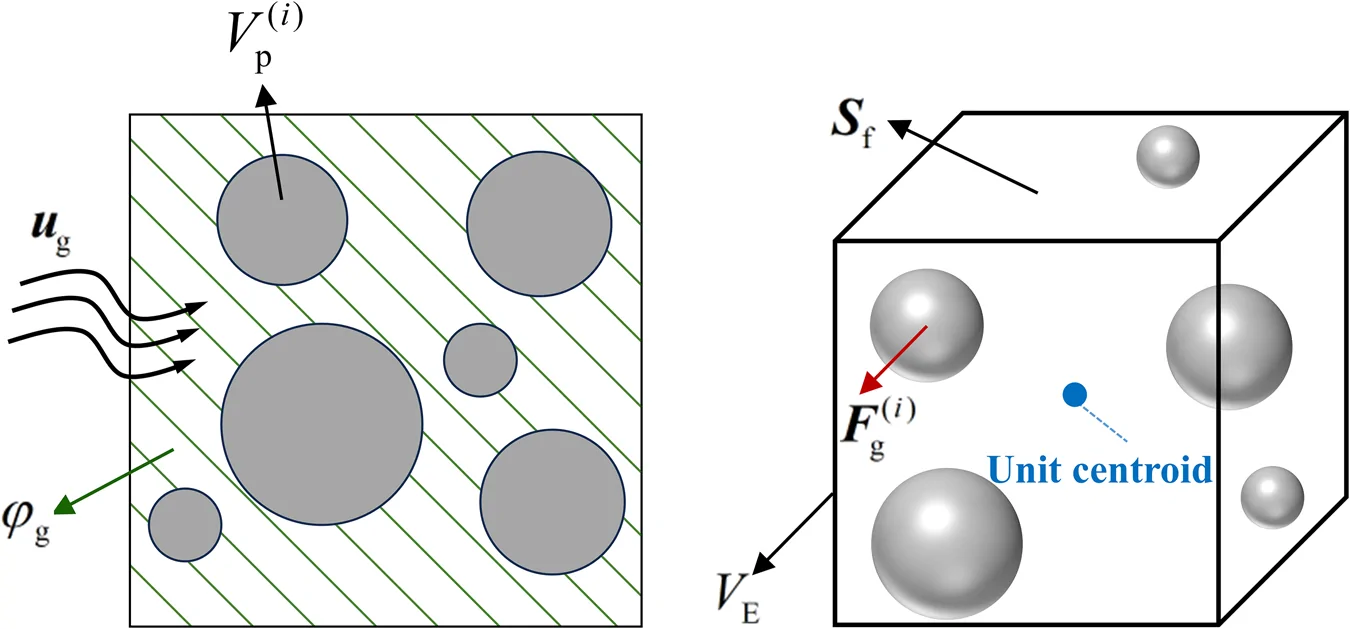
Schematic diagram of particle fluid force calculation in the fluid unit.

(2) Conservation equation of momentum [27]:

(2.3) $$\frac{\partial }{\partial t}\left(\varphi_{g}\rho_{g}u_{g}\right)+\nabla \times \left(\varphi_{g}\rho_{g}u_{g}⊗u_{g}\right)=-\varphi_{g}\nabla p_{g}+\varphi_{g}\nabla \times \vec{\vec{\tau }}+S_{f}+\varphi_{g}\rho_{g}g,$$

where $\vec{\vec{\tau }}$ is the viscous stress tensor of fluid, *S_f_* is the momentum exchange force source term between the fluid and the particle [28] and *g* is the gravitational acceleration. Detailed definitions of *φ*_*g*_ and *S_f_*, such as equation (2.3), figure 2 is the schematic diagram.

(2.4)φg=1−φP=1−1VE∑i=1nP⁡VP(i)(2.5)andSf=1VE∑i=1np⁡Fg(i),

where *V_E_* is the volume of fluid unit, *φ*_*P*_ is the volume fraction of particles in the fluid unit, $V_{P}^{\left(i\right)}$ is the volume of the first *i*-th particle in the fluid unit, $\;F_{g}^{\left(i\right)}$ is the *i*-th particle in the fluid unit subjected to fluid force and *n_P_* is the number of particles in the fluid unit.

(3) Component transport equation [29]:

(2.6) $$\varphi \frac{\partial }{\partial t}\left(\rho_{i}\omega_{i}\right)+\nabla \times \left(\rho_{i}\omega_{i}u_{i}\right)=\nabla \times \left(\rho_{i}D\nabla \omega_{i}\right),$$

where *ρ*_*i*_ is the gas density corresponding to component *i*, *ω*_*i*_ is the mass concentration corresponding to component *i* and *D* is the diffusion coefficient.

(4) When the pipeline leakage occurs, the gas flow at the leak port becomes turbulent under the action of a huge pressure difference inside and outside the pipeline. Compared with the standard *k*-*ε* model, the Realizable *k*-*ε* model demonstrates superior predictive capability for complex flows involving significant pressure gradients and high-speed jets, providing a more accurate depiction of the intense shear and turbulent diffusion phenomena generated by the high-speed gas ejection near leakage holes [30]. Therefore, the Realizable *k*-*ε* model is used to describe turbulent flow.

(2.7)∂∂t(ρk)+∂∂xi(ρkui)=∂∂xi[(μ+μtprk)∂k∂xi]+Gk+Gb−ρε−YM+SK(2.8)and∂(ρε)∂t+∂(ρεui)∂xi=∂∂xi[(μ+μtprε)∂ε∂xj]+ρC1Sε−ρC2ε2k+υε+C1εkεkC3εGb+Sε.

Wherein:

(2.9) $$C_{1}=max\left[0.43,\frac{\eta }{\eta +5}\right],\eta =S\frac{k}{\varepsilon },S=\sqrt{2S_{ij}S_{ij}},$$

where *G_k_* is the turbulent kinetic energy generation term caused by the average velocity gradient, *G_b_* is the turbulent kinetic energy generation term caused by buoyancy, *Y_M_* is the increase in total dissipation rate caused by pulsating expansion in compressible turbulence, *C*_2_ and *C*_1*ε*_ are constants, *σ*_*k*_ and *σ*_*ε*_ are the Turbulent Prandtl numbers of *k* and *ε* and *S_ij_* is the source term in different directions.

#### 2.2.2. Particle motion equation

(1) Particle force analysis

(2.10) $$F_{g}=F_{d}+F_{P}+F_{vm}+F_{saff}+F_{ml}+F_{b},$$

where *F_d_* is the resistance (drag force) that particles experience in the fluid, *F_P_* is the pressure gradient force, *F*_vm_ is the virtual mass force [31], *F*_saff_ is the Saffman shear lift [32], *F*_ml_ is the Magnus rotational lift [33] and *F_b_* is the Basset force [34]. Owing to the small particle size, resistance (drag force) plays the main role in the force exerted by the fluid on the particles, ignoring other forces [35]. Additionally, since the fluid velocity at the leakage port is relatively fast, the gas velocity should be greater than the particle velocity and the force acting on the particles is manifested as drag force. The calculation formula [36] is:

(2.11) $$F_{d}=\frac{1}{8}C_{d}\pi D_{P}^{2}\left|u_{g}-u_{P}\right|\left(u_{g}-u_{P}\right),$$

where *D_P_* is the particle diameter and *D_d_* is the drag coefficient, calculated using the Gidaspow model [37].

(2) Selecting any particle *i* as the analysis object within the fluid unit, its motion is mainly composed of particle translation and particle rotation. The following equation [38] is used to describe it:

(2.12)mPduPdt=(Fc,n+Fc,t+Fa)+Fg+mPg(2.13)andIPdωpdt=Mt+Mg+Mr,

where *F*_*c*,*n*_ is the normal contact force, *F*_*c*,*t*_ is the tangential contact force, *F_a_* is the adhesion between particles; *M_t_* is the tangential contact torque of particles, *M_g_* is the torque generated by the fluid velocity gradient on the particles and *M_r_* is the rolling torque.

The Hertz–Mindlin contact model [39] is used to describe *F*_*c*,*n*_ and *F*_*c*,*t*_, *F*_*c*,*n*_ is mainly composed of elastic and viscous forces, and its definition [40] is:

(2.14)Fc,n=Fe+Fv,(2.15)Fe=−43ERδn3/2(2.16)andFv=Cnδn⋅,

where *E* is the equivalent Young's modulus of the contact pair, *R* is the equivalent radius of the contact pair, *δ*_*n*_ and $\dot{\delta_{n}}$ are the normal overlap and normal relative velocity of the contact pair and *C_n_* is the normal damping coefficient.

The definition of tangential contact force *F*_*c*,*t*_ [41] is:

(2.17) $$F_{c,t}=min\left(\left|K_{t}\delta_{t}-C_{t}\overset{\cdot }{\delta_{t}}\right|,\mu \left|F_{c,n}\right|\right)t,$$

where *δ*_*t*_ and $\dot{\delta_{t}}$ are the tangential relative displacement and tangential relative velocity of the contact pair, µ is the equivalent friction coefficient of the contact pair, *t* is the tangential direction vector, *K_t_* and *C_t_* are the tangential stiffness and tangential damping coefficient, respectively.

The adhesion force *F_a_* between particles is defined using the Johnson–Kendall–Roberts contact model [42]:

(2.18) $$F_{a}=4\sqrt{\pi \alpha^{3}\gamma E},$$

where *γ* is the surface energy and *α* is the radius of the contact surface.

The definitions of each torque are as follows [43]:

(2.19)Mt=DP2n×Fc,t,(2.20)Mg=ρf2DP2CR5|12ωf−ωP|(12ωf−ωP)(2.21)andMr=−μrrPFc,nωP|ωP|,

where *ω*_*f*_ is the fluid angular velocity vector, *C_R_* is the rotation coefficient, calculated from the Reynolds number, *ω*_*P*_ is the particle angular velocity vector, µ_*r*_ is the rolling resistance coefficient and *r_P_* is the rolling radius of particles.

#### 2.2.3. CFD–DEM coupling

By analysing the fluid motion control equations in the first part and the particle motion control equations in the second part of this section, it can be concluded that the interaction between fluid and particles can be achieved through data exchange. Based on CFD calculation of *φ*_*g*_ and *S_f_* to solve the fluid motion control equations, and based on DEM calculation of *F_g_* and *M_g_* to solve the particle motion control equations.

### 2.3. Mesh generation and independence verification

In CFD–DEM simulation, the mesh is the medium of momentum and energy exchange between fluid and particles, and its division strategy will directly affect the accuracy and efficiency of the solution. The unstructured grid is used to discretize the computational domain, and the leakage port is locally densified to improve the capture ability of the flow field changes in the high-speed gas leakage area. In addition, the boundary layer is added to the pipe wall. The overall mesh size is about 1–1.5 times the average diameter of particles, which can ensure the calculation accuracy and reduce the calculation cost. The schematic diagram of mesh division is shown in figure 3.

**Figure 3.**
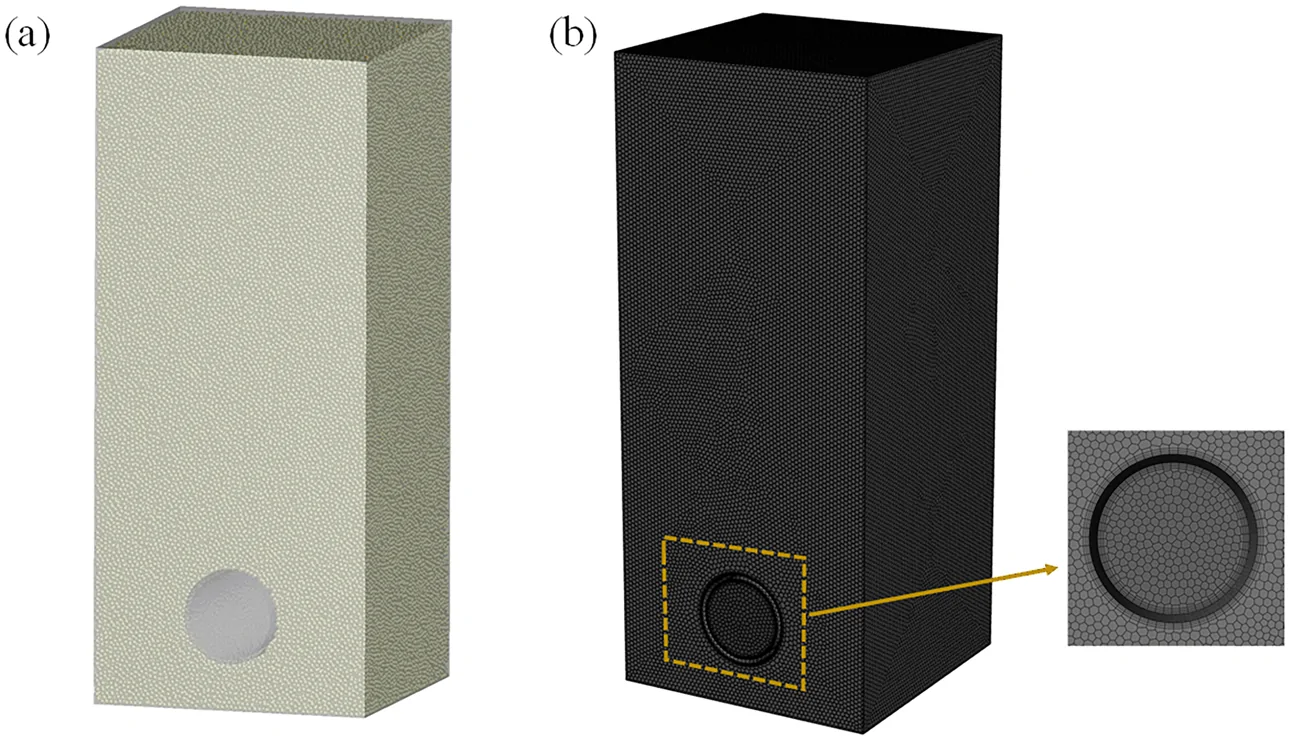
Schematic diagram of grid division.

To verify the influence of grid division on the calculation results, four different numbers of grids are divided and the maximum gas leakage velocity under the four grid numbers is compared and analysed, as shown in table 2. It can be seen from table 2 that with the increase of the number of grids, the difference between the maximum gas leakage rates under different division numbers gradually tends to be stable and the error is within 3%. To save computing costs, 265 984-quantity division scheme is selected for subsequent grid division.

**Table 2.** Grid independence verification.

number of mesh	maximum leakage rate (kg s^−1^)	relative error (%)
58475	0.13221	—
112584	0.14631	10.67
265984	0.14985	2.42
452120	0.15341	2.38

### 2.4. Simulation condition setting

In this paper, the coupling method of CFD–DEM is adopted to study the soil structure deformation behaviour and gas leakage diffusion after the leakage of a buried gas pipeline. The CFD calculation is carried out by using ANSYS fluent 2022, with the use of a transient pressure based solver and coupled with the coupled algorithm to solve for pressure and velocity and the convection term is discretized by using the second-order upwind scheme, the time step is set to 1 × 10^−5^ s, Altair EDEM is selected for DEM solution and the middle time step is one tenth of the corresponding step of CFD, which is 1 × 10^−6^ s, to meet the requirements of stable particle contact. The data exchange between CFD and DEM is carried out every 10 steps to realize the bidirectional coupling between the flow field and particle motion.

In the CFD boundary condition setting, the pipeline inlet is set as the pressure inlet, the pipeline outlet and soil surface are set as the pressure outlet, both the inner and outer walls of the pipeline are set as wall surfaces and the leakage port on the pipe wall and the soil fluid domain are set as internal. In the DEM boundary condition setting, the pipe wall and soil surface are set as wall surfaces, and particle filling is used for the soil fluid domain. The specific boundary conditions are shown in table 3.

**Table 3.** Type of boundary conditions.

boundary	CFD type	DEM type
pipeline inlet	pressure inlet	-
pipeline outlet	pressure outlet	-
the pipe wall	wall surface	wall surface
soil boundaries	pressure outlet	wall surface
soil zone	Interior	particle filling

### 2.5. Reliability verification of CFD–DEM simulation method

To verify the reliability of the CFD–DEM coupling model in this paper, the same theoretical verification method is used as in Li, and the seepage field model based on Darcy's law is selected for comparison. To establish the same seepage model as Li, maintain consistency of soil particle size, porosity, fluid density, fluid viscosity and other parameters, the calculation domain size is 80 × 20 cm^2^, we set the same boundary conditions and simulate the seepage flow under these conditions. The calculation of seepage flow is shown in figure 4. It can be seen from the figure that the theoretically calculated seepage flow is 9.52 × 10^−6^ m^3^ s^−1^, the simulated seepage flow of Li is 9.09 × 10^−6^ m^3^ s^−1^ and the calculated simulated seepage flow of this paper is 8.95 × 10^−6^ m^3^ s^−1^. The error between the simulated result and the theoretical calculation is 5.9%, which is consistent with the overall change trend of Li's research and the average relative error is 2.4%. Therefore, it can be considered that the CFD–DEM model used in this paper can better describe the migration process of soil particles. The error can be attributed to the difference in local pore structure caused by the random generation of particles in DEM and is also related to the differences in mesh generation and time-step setting in CFD.

**Figure 4.**
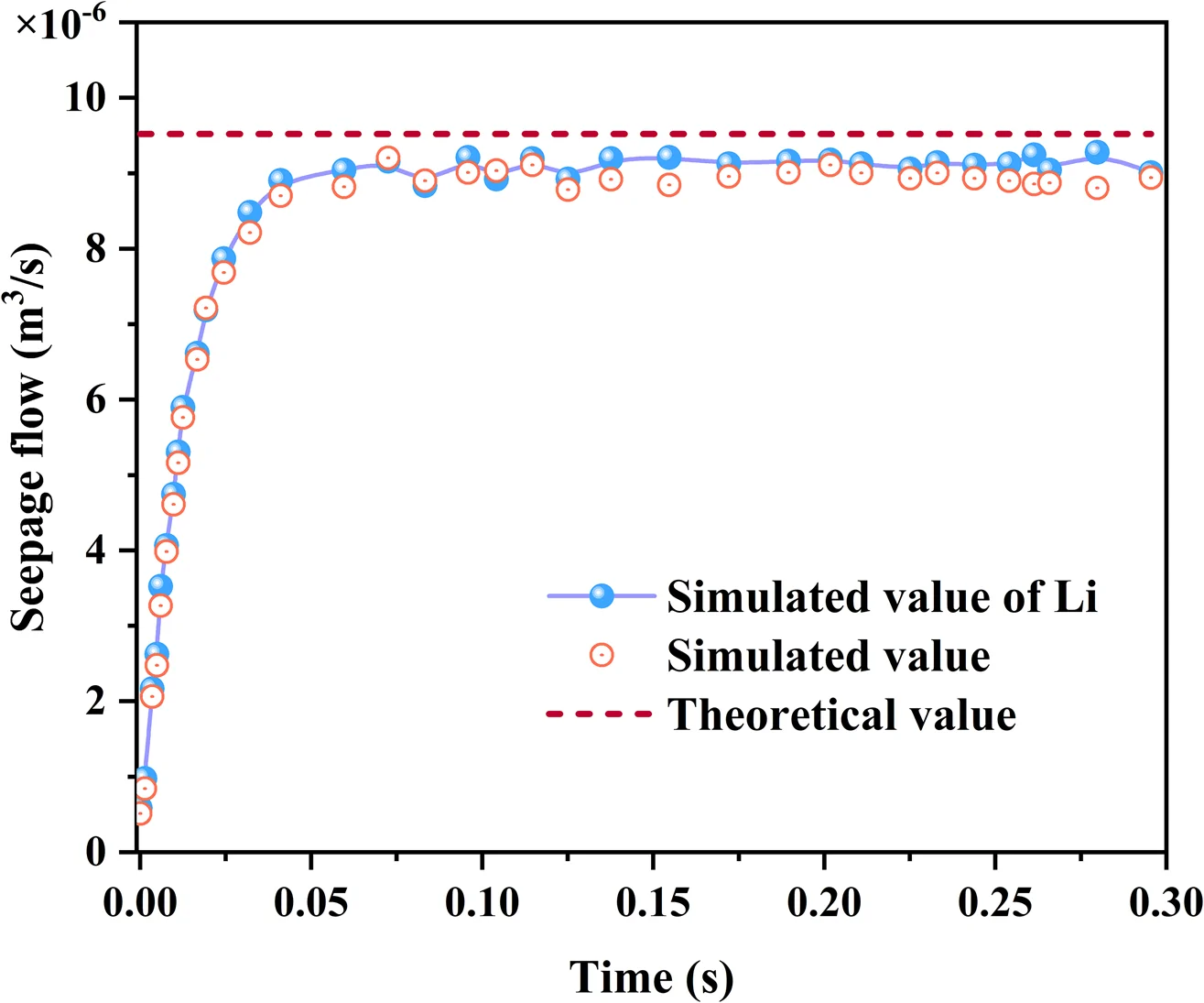
Model reliability test.

### 2.6. Case parameter setting

The research object of this paper is the leakage of a medium-pressure buried gas pipeline. The soil particle diameter, porosity, moisture content and pipeline operating pressure are taken as research variables, and the control variable method is used to explore the influence of the above factors on the soil cavity and gas leakage diffusion. Among them, different soil porosities are achieved by controlling the number of particles filled in the soil domain, using equation (2.22) to calculate the corresponding number of particles; the effect of changes in soil moisture content on mechanical properties [44] is approximately characterized by setting particle contact parameters. Among these, the shear modulus, static friction coefficient and dynamic friction coefficient of particles are mainly considered, but the capillary bridge force, matrix suction and liquid migration are not considered. Therefore, the applicability to highly saturated soil is still limited. In order to reduce the error caused by this, this paper only considers cases where the water content is less than 30%. The corresponding settings for different moisture contents are shown in table 4.

**Table 4.** Particle physical parameter setting.

moisture content	shear modulus (MPA)	coefficient of static friction	coefficient of rolling friction
10%	8.114	0.832	0.139
20%	7.839	0.649	0.157
30%	6.138	0.552	0.288

Detailed settings for each working condition are shown in table 5. Working condition 2 is selected as the basic working condition, in which the particle diameter, soil porosity, soil moisture content and pipeline operating pressure are set to the medium level of the parameter range for each working condition, so as to avoid the interference of a single parameter being too high or too low on the regularity analysis of the research results.

(2.22) $$N_{P}=\frac{\left(1-\varphi \right)V}{\frac{4}{3}\pi D_{P}^{3}},$$

where *N_P_* is the number of particles, *φ* is the soil porosity and *V* is the soil volume.

**Table 5.** Case conditions.

case	particle diameter (mm)	porosity	moisture content	pipeline pressure (MPa)
1	4	0.45	0.2	0.3
2	5	0.45	0.2	0.3
3	6	0.45	0.2	0.3
4	5	0.4	0.2	0.3
5	5	0.5	0.2	0.3
6	5	0.45	0.1	0.3
7	5	0.45	0.3	0.3
8	5	0.45	0.2	0.2
9	5	0.45	0.2	0.4

## 3. Results and discussion

### 3.1. The influence of soil deformation behaviour on gas leakage and diffusion

Taking case 2 as the standard working condition, this study analysed the pressure field, velocity field, leakage gas streamlines and soil particle movement within 0.1 s after the leakage of the buried gas pipeline, and explored the interaction and connection between soil deformation behaviour and leakage gas diffusion.

#### 3.1.1. Pressure field analysis

To analyse the pressure distribution inside the soil after the leakage of buried gas pipelines, the pressure distribution inside the soil sliced along the x-axis direction after 0.1 s of leakage is shown in figure 5. Figure 5a shows that the overall pressure distribution in the soil is in the shape of a ‘light bulb’ after the occurrence of a buried gas pipeline leak, and the closer the pressure is to the leakage port, the higher the pressure value. Figure 5b shows the pressure distribution near the leakage port, and the contour indicates that the pressure value in the soil rapidly decreases with the increase of diffusion distance. The pressure value at 0.1 m directly above the leak is between 100 and 500 Pa. When the distance increases to 0.2 m, the pressure value drops to between 5 and 10 Pa, which is very close to the atmospheric pressure. At this time, the change in the pressure field caused by the leak can be ignored. It can be explained that the pressure value has a nonlinear relationship with the diffusion distance, and the rate of decrease in pressure value far exceeds the rate of increase in diffusion distance. If the pressure area above 1000 Pa is considered as the effective pressure influence range caused by leakage, then the effective pressure influence range of leakage is concentrated in the area 0.05 m directly above the leakage port. It is worth noting that the corresponding pressure values on both sides of the effective pressure influence range are negative; this is because when the gas leaks from the high-pressure area to the low-pressure soil, the local flow rate increases rapidly. According to Bernoulli's principle, the increase in flow rate will lead to a reduction in local static pressure. At the same time, the soil hinders the gas flow, limiting the timely supplement of the surrounding air to the leakage area [45,46].

**Figure 5.**
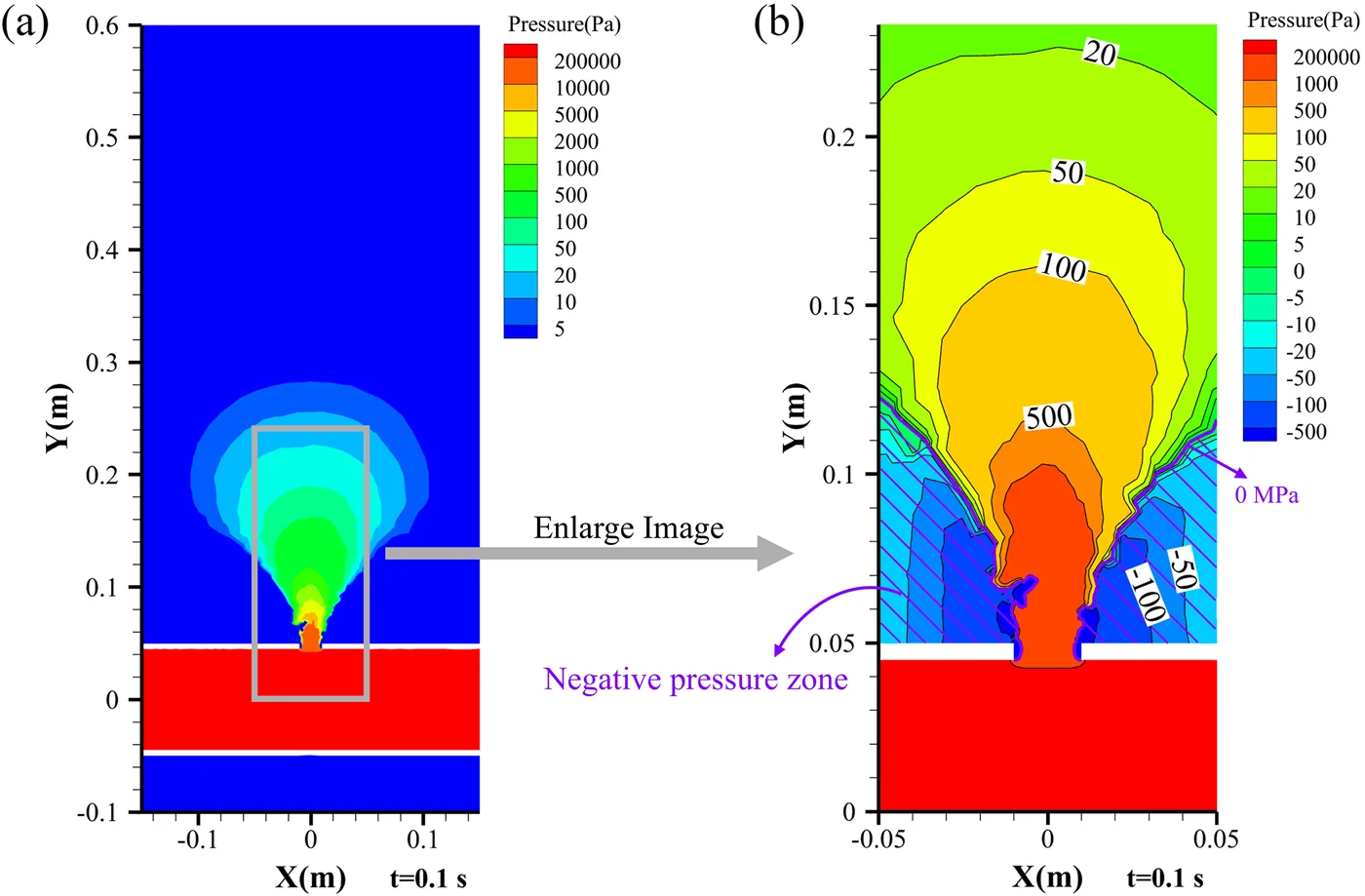
Pressure contour.

Figure 6 shows the pressure changes at each monitoring point in the soil. As monitoring points A, B and 1 are within the influence range of effective pressure, the corresponding pressure values reach more than 1000 Pa, which is much higher than that of other monitoring points; this is consistent with the law shown in the pressure contour. Within 0.005 s before the leakage, the pressure value of each monitoring point fluctuated significantly, and then the pressure of each point tended to be stable. But at 0.053 s after the leakage occurred, the pressure value at monitoring point 1 showed significant fluctuations again, probably owing to the sudden change in pressure caused by the movement of soil particles. Observing the stable pressure values at each point, it can be found that except for the three monitoring points within the effective pressure influence range, the corresponding pressure values at all other monitoring points are around atmospheric pressure, and the pressure values of monitoring points 2 and 4 are about −20 Pa, which is consistent with the law of the negative pressure zone shown in the pressure contour. The instantaneous release of gas compresses the pore space of the surrounding soil, resulting in negative pressure on both sides of the leakage hole. After 0.01 s, the pressure at the monitoring point gradually returns to normal. This is because at this time, the soil particles are rearranged, voids begin to form and the priority path of gas migration is determined.

**Figure 6.**
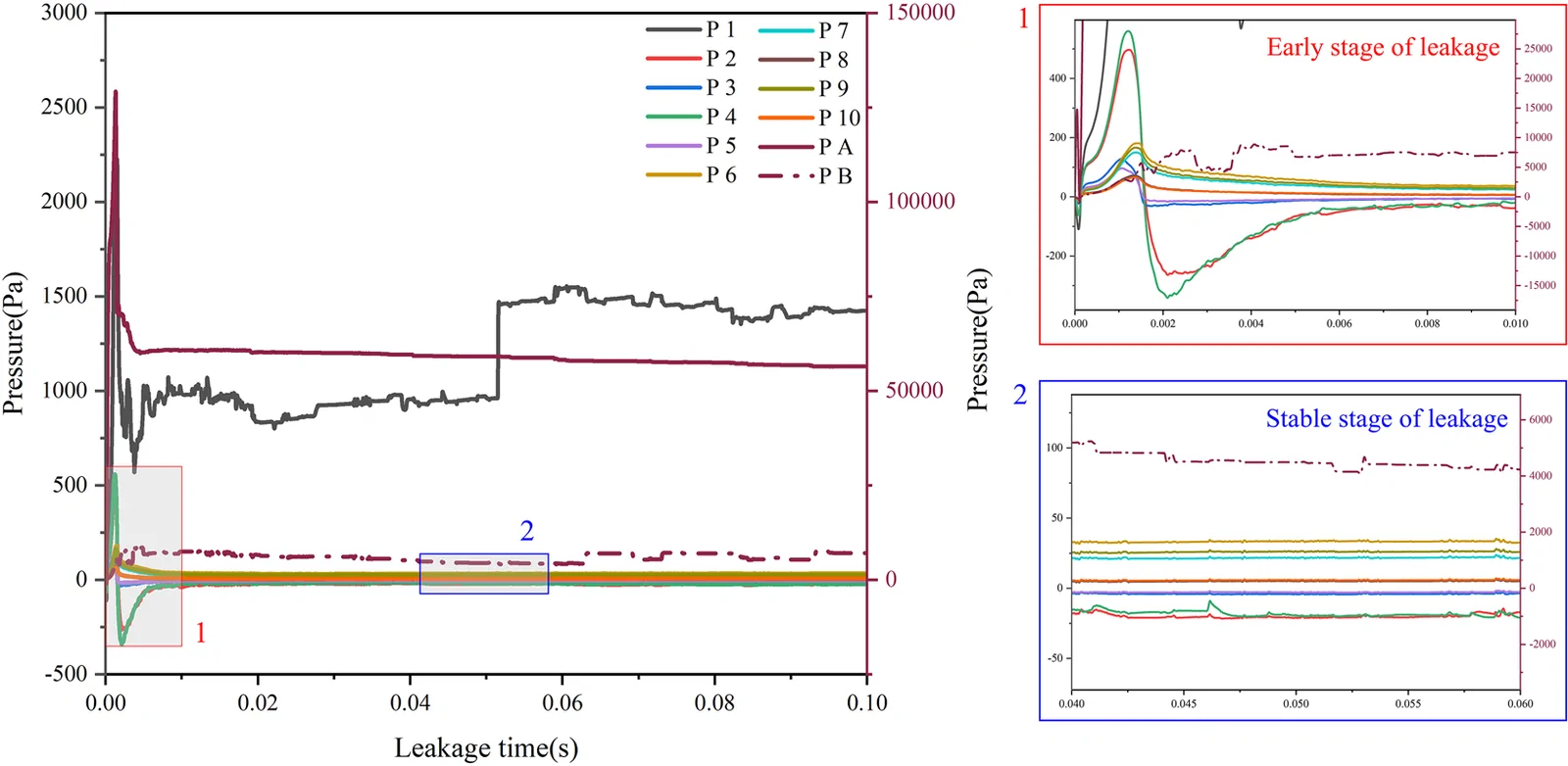
Monitoring point pressure changes.

#### 3.1.2. Velocity field analysis

To analyse the velocity distribution inside the soil after the leakage of buried gas pipelines, the velocity distribution inside the soil sliced along the x-axis direction after 0.1 s of leakage is shown in figure 7. Figure 7a shows that after the leakage of the buried gas pipeline, not only the velocity field in the soil is significantly affected, but also the velocity field inside the pipeline at the leakage port also changes. Owing to the huge pressure difference inside and outside the pipeline, the velocity value at the leakage port is extremely high, and the maximum velocity value reaches more than 800 m s^−1^. Supersonic leakage is often found in leakage incidents of overhead pipelines, and the leakage velocity contour of an overhead pipeline is usually in the shape of a jet [14]. However, owing to the resistance of the soil covering the buried gas pipeline to the leakage gas, the leakage velocity contour presents a ‘wick’ shape, and with the increase of the distance from the leakage port, the velocity value decreases greatly, and the high-speed area is mainly concentrated near the leakage port. Figure 7b shows the velocity distribution near the leakage port, which is consistent with the pressure gradient change trend presented in the pressure contour. The decreasing speed of the velocity value far exceeds the increasing speed of the diffusion distance, and the high-speed zone in the velocity contour is concentrated within 0.05 m directly above the leakage port, which corresponds to the effective pressure influence range in the pressure contour. It indicates that the negative pressure zone on both sides of the effective pressure influence range in the pressure contour is owing to the pressure drop on both sides of the fluid owing to high-velocity flow.

**Figure 7.**
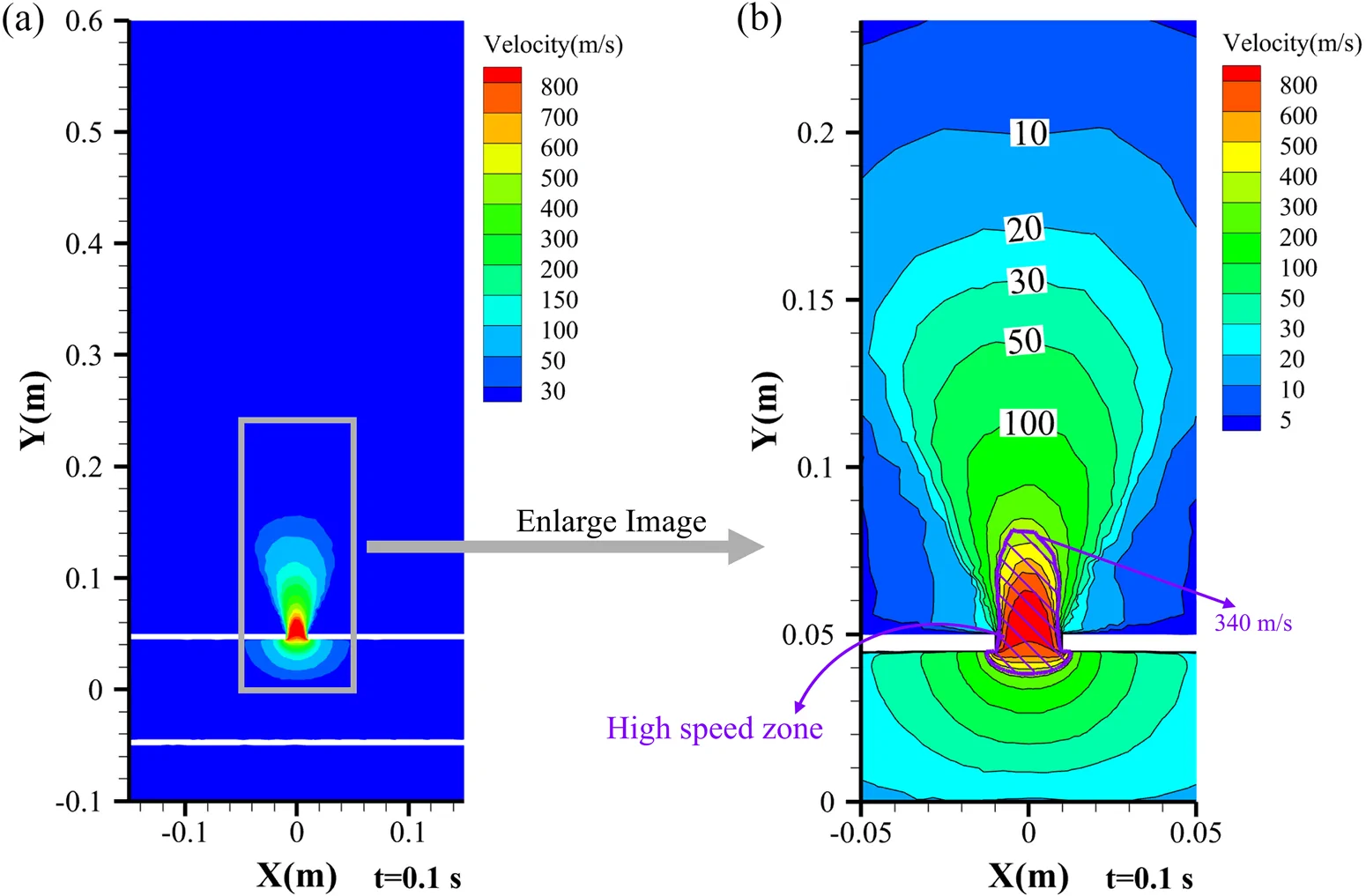
Velocity contour.

Figure 8 shows the pressure changes at each monitoring point in the soil. The velocity corresponding to monitoring points A, B and 1 is far higher than that of other monitoring points, especially the velocity values of monitoring points A and B exceed 850 and 350 m s^−1^, respectively. The leakage speed at monitoring point 1 is also at a relatively high value, exceeding 125 m s^−1^. Within 0.005 s after leakage, the velocity values of each monitoring point fluctuate significantly, and the subsequent velocity values of each point tend to be stable. In addition to the above three monitoring points, the speed values of the remaining monitoring points are kept in a low range. Among them, the velocity corresponding to monitoring point 6 is the largest, exceeding 8 m s^−1^, and the velocity value corresponding to monitoring point 3, which is closer to the leakage port in a straight line, is less than 2 m s^−1^, indicating that the velocity field above the leakage port is more affected by pipeline leakage, and the diffusion movement of leaking gas is dominated by the vertical direction and accompanied by some horizontal diffusion.

**Figure 8.**
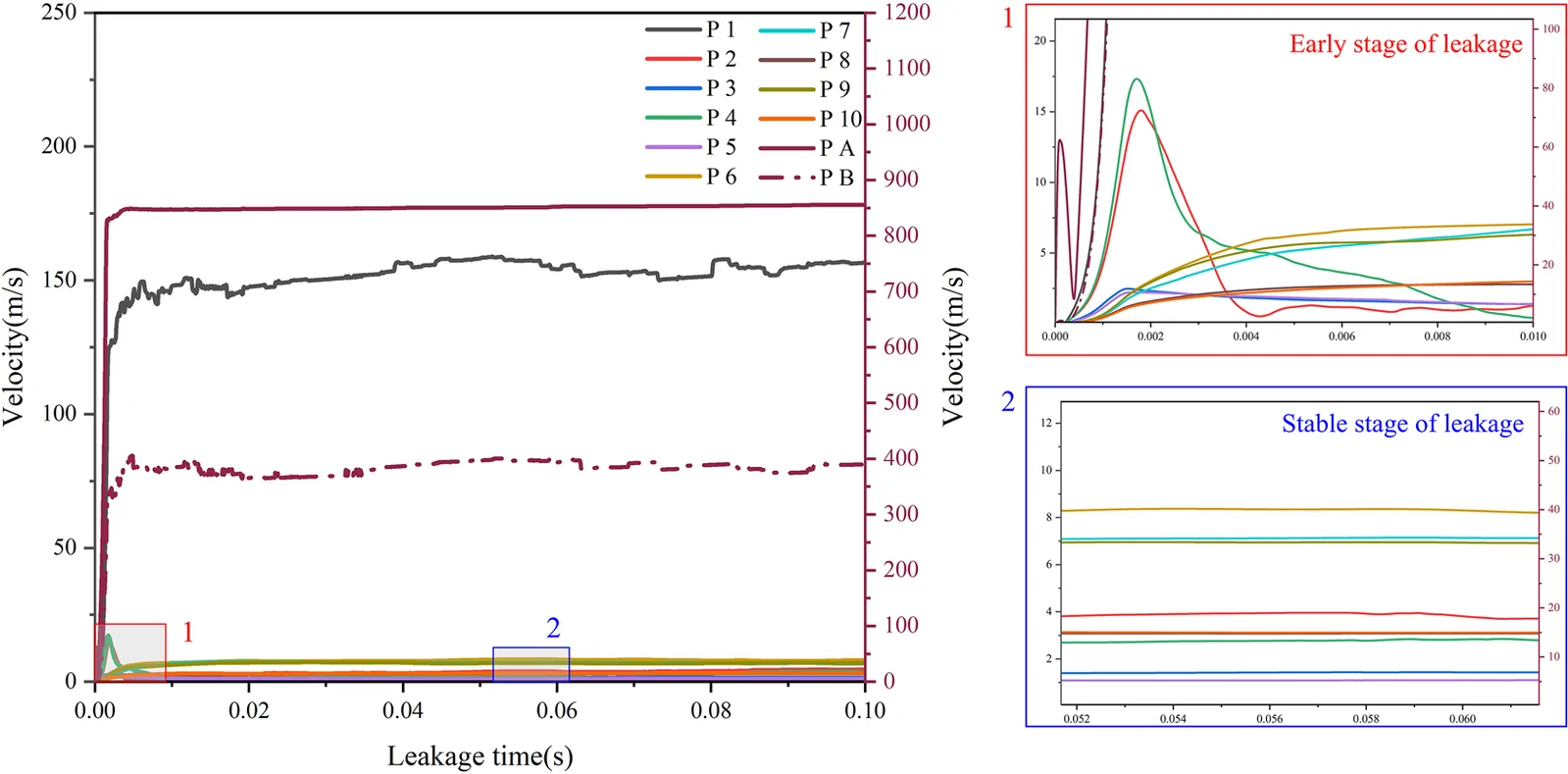
Monitoring point velocity changes.

#### 3.1.3. Streamline analysis

To understand the leakage gas movement inside the soil after the leakage of the buried gas pipeline, the streamline distribution inside the soil after the leakage of the pipeline was analysed. Figure 9 shows the streamline distribution along the x-axis direction at different times. As shown in the figure, the maximum vertical heights of gas movement at *t* = 0.005, 0.01, 0.05, and 0.1 s are 0.445, 0.426, 0.392 and 0.379 m, respectively. The maximum height gradually decreases, with the rate of decrease also diminishing. In addition, with the increase of leakage time, the range of streamlines in the horizontal direction increases. This shows that the original unidirectional flow of the gas in the pipeline has changed into the movement of the gas on both sides of the leakage port. Under the effect of the pressure difference between the inside and outside of the pipeline, the leakage gas is sprayed into the soil from the leakage port. Owing to the resistance of the soil to the leakage gas, the movement of the leakage gas in the vertical direction has declined, and the vertical upward diffusion has gradually changed to horizontal diffusion. At the same time, a large amount of high-speed gas movement at the leakage port drives the airflow in the soil, resulting in eddy currents on both sides of the leakage port.

**Figure 9.**
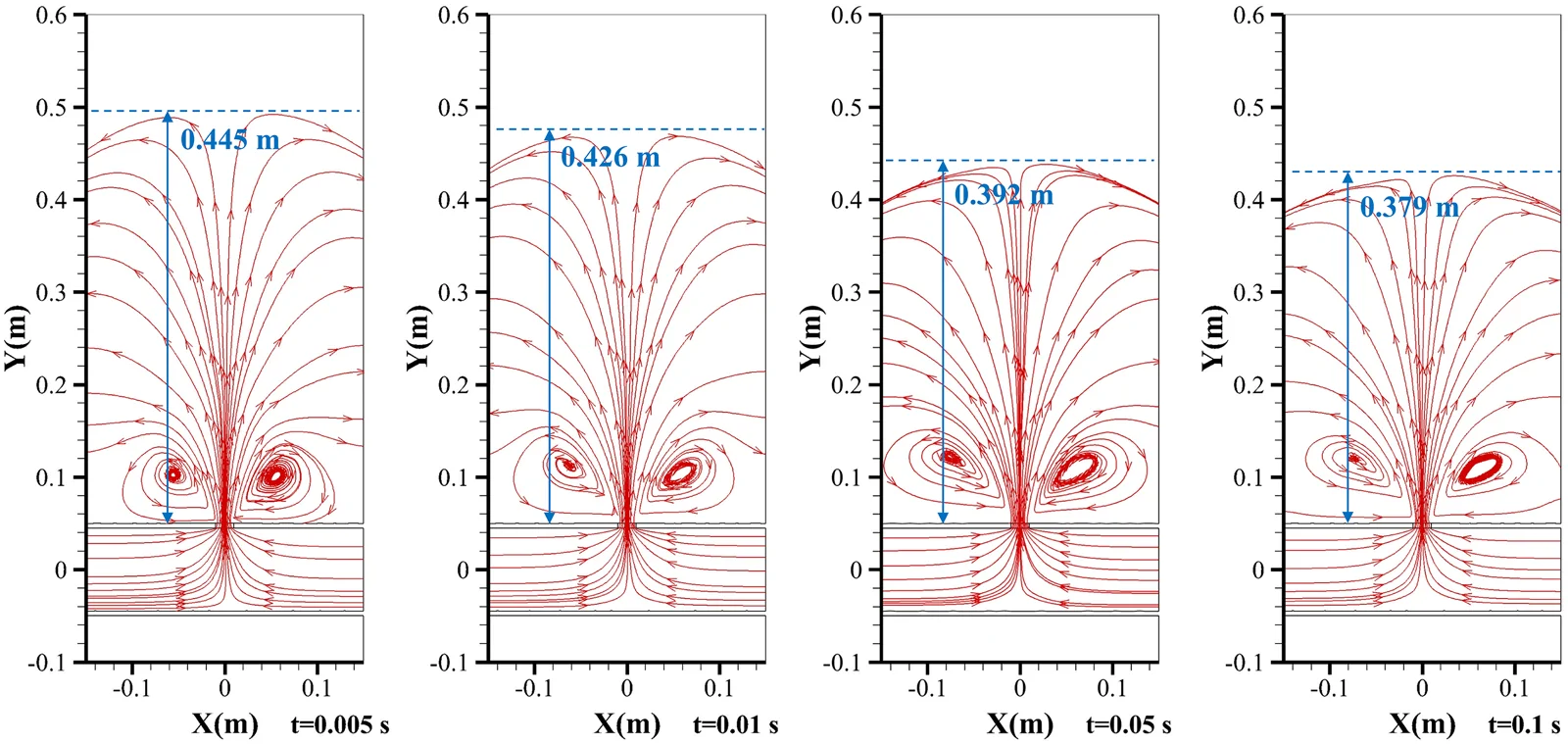
The gas streamlines distribution in the soil.

#### 3.1.4. Soil cavity formation process and methane concentration distribution

Figure 10 shows the movement of soil particles near the leakage port following the pipeline leak. As shown in the figure, when the pipeline leakage occurs for 0.003 s, the soil particle movement speed is high, at this time, the soil structure has changed greatly. According to the analysis of the velocity field inside the soil in §3.1.2, the velocity value at the monitoring point A at this time has exceeded 850 m s^−1^. Under the impact of high-speed airflow, the motion speed of soil particles near the leakage port exceeds 1 m s^−1^. When the pipeline leakage occurs for 0.005 s, the number of soil particles moving at high speeds began to decrease and the velocity of most of the particles dropped to below 0.5 m s^−1^. When the pipeline leakage occurs for 0.01 s, most of the soil particles had already stabilized, and a few particles were still in motion, while when the pipeline leakage occurs for 0.1 s, the soil particles above the leakage port were no longer in motion, and the leakage gas impact led to the soil cavity being in a stable state. Quantitative analysis was conducted on the size of soil cavity at this time, when extracting the maximum height and width of the cavity, considering that the actual cavity edge has certain irregularity owing to the random arrangement of discrete particles, in order to facilitate the subsequent geometric characterization, the shape of the cavity edge is idealized, that is, its contour is approximately regarded as a regular arc shape. At this time, the maximum height of the cavity is 10.70 mm and the maximum width is 23.26 mm. Figure 11 shows the soil particle velocity curve. The maximum particle velocity curve indicates that the particle movement velocity reaches its maximum value at 0.002 s, exceeding 5 m s^−1^. After reaching the peak velocity, it begins to decrease and tends to stabilize. At 0.1 s, the maximum particle velocity is already less than 0.05 m s^−1^, indicating that the soil particles have stabilized. The trend of the average particle velocity curve is consistent with that of the maximum speed curve, rapidly rising to the peak and then decreasing, eventually stabilizing. The analysis of soil particle movement near the leakage port shows that the leakage gas causes the soil particles near the leakage port to collide and move rapidly in a very short time, and the formation speed of the soil cavity is extremely fast. After 0.1 s of pipeline leakage, the soil cavity has stabilized. This is consistent with the research rule of Li *et al.* [24]. After the leakage, the particles will migrate rapidly to the leakage hole and form a local cavity above the leakage hole after stabilization.

**Figure 10.**
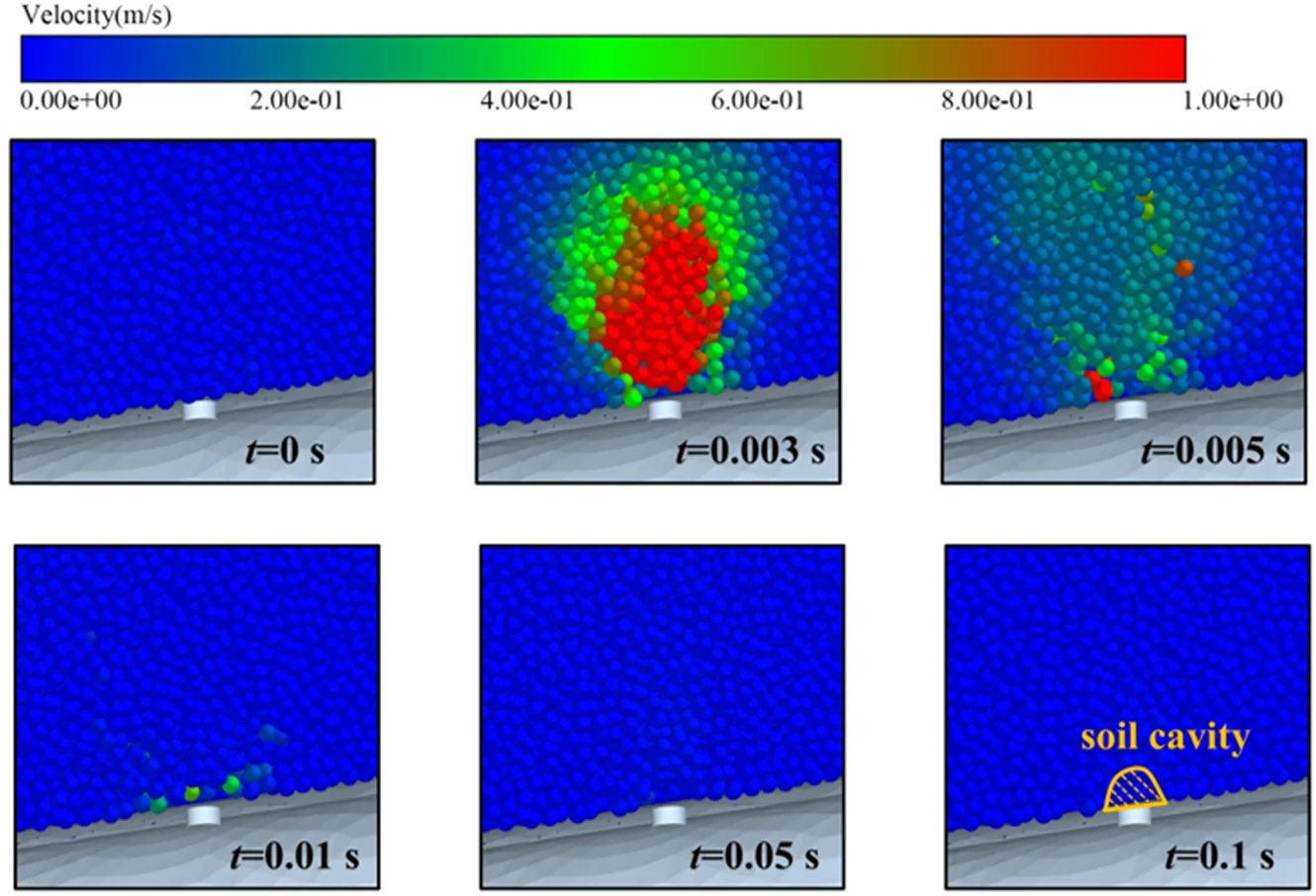
The process of soil cavity formation.

**Figure 11.**
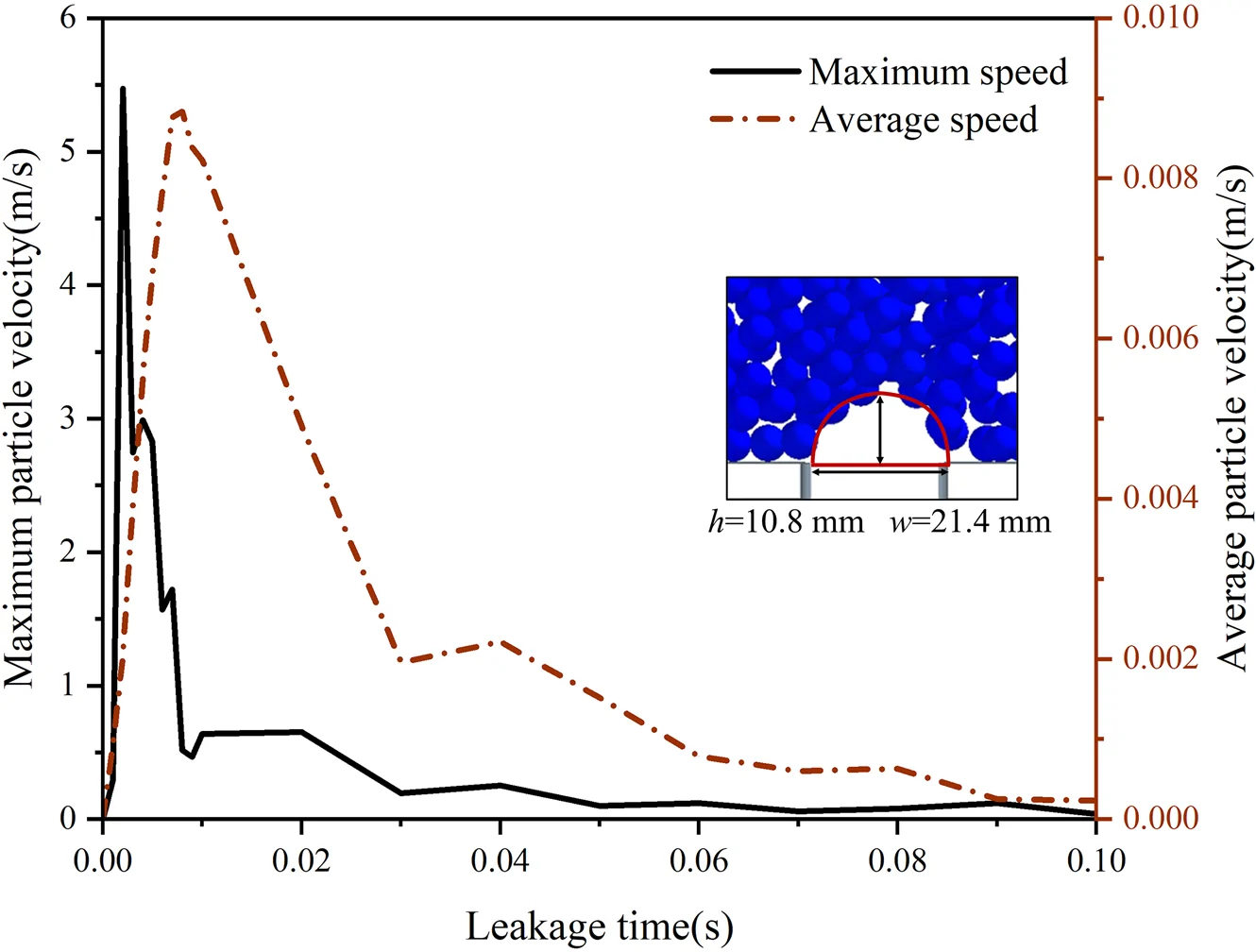
Particle velocity variation.

Figure 12 shows the distribution of methane concentration in slices along the x-axis direction at different time points, and the contour shows that methane diffuses in the soil in a circular shape. Analysing the high-concentration zone where methane concentration is above 90%, the diffusion profile of the high-concentration zone shows a vertical jet, which is consistent with leakage from the overhead pipeline, owing to the rapid diffusion of the leaking gas caused by the great pressure difference between the inside and outside of the pipeline. But after being subjected to soil resistance, methane began to diffuse horizontally around the soil, indicating that the soil not only slowed down the diffusion velocity of methane, but also changed the direction of methane diffusion. This diffusion characteristic is mainly related to the changes in gas migration mechanisms in different regions. There is a strong pressure gradient at the leakage port, and the gas migration in this area is mainly characterized by pressure-driven convection. As the diffusion distance increases, the gas is affected by soil resistance and the pressure gradient decreases continuously. At this point, the contribution of molecular diffusion driven by the concentration gradient is enhanced and cannot be ignored. To further verify this process, extract the velocity on monitoring line 1 and estimate the Péclet number. The results show that at a distance of 0.05 m near the leakage hole, the Péclet number is much greater than 1, indicating that convection dominates absolutely at this time; At a distance of 0.2 m above the leakage hole, the flow velocity drops to 8 m s^−1^, and at this point, the Péclet number is about 8 × 10^4^, still exhibiting convective-dominant characteristics. When the distance further increases to 0.5 m, the flow velocity has dropped to the order of 10^−3^ m s^−1^, and the Péclet number is about 20. At this point, molecular diffusion cannot be ignored. This estimation result is consistent with the variation of the methane diffusion law, that is, a clear vertical high-speed jet is formed in the early stage of leakage, and as the momentum continues to decay, the gas gradually diffuses towards the surrounding area.

**Figure 12.**
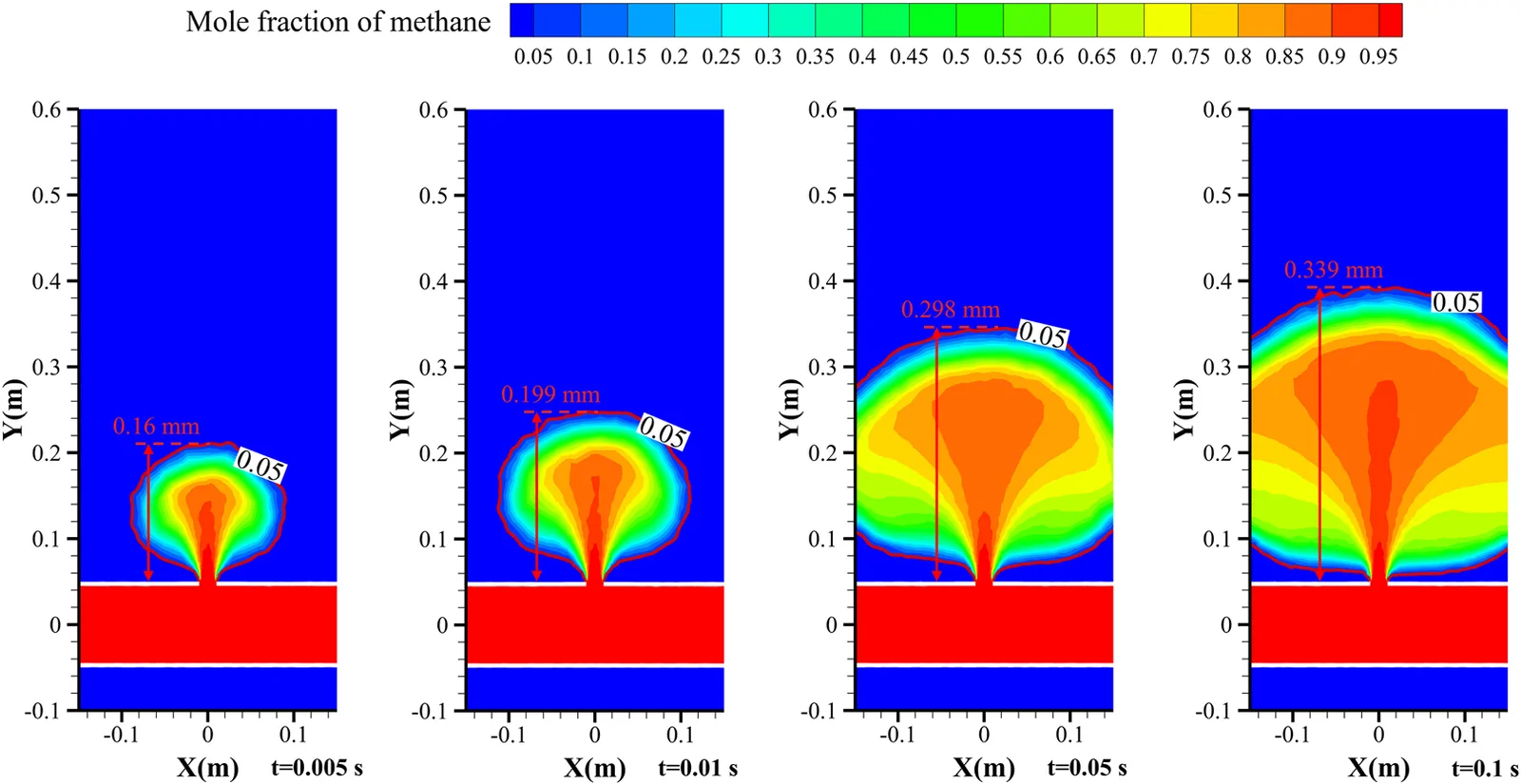
Methane concentration contour.

The explosion caused by the leakage of buried gas pipelines is one of the main hazards of gas pipeline accidents. The red curve in the contour reflects the explosion range of methane in soil (5% concentration is the lower limit concentration of methane explosion [47]), and the highest dangerous range (HDR) of methane vertical diffusion in soil, which is the highest range when methane concentration reaches the lower explosive limit, is used as the subsequent evaluation index for methane diffusion in this study. The contour shows that the HDR increases as the leakage time increases.

### 3.2. The influence of soil particle size on leakage process

#### 3.2.1. Different particle diameters correspond to soil cavity comparison

Figure 13 shows the size of soil cavity corresponding to different soil particle diameters after pipeline leakage occurred for 0.1 s. As shown in the figure, the porosity corresponding to different soil particle diameters is consistent, all of which are 0.45. However, with the increase in soil particle diameter, the soil pore structure in the soil undergoes significant changes, and the soil pore size corresponding to the soil particle diameter of 6 mm is larger. This indicates that the change of soil particle size will affect the pore structure inside the soil under the same porosity. The smaller the diameter of soil particles, the more detailed the filling of the soil domain, and the corresponding soil pores are more dispersed and finer, while the larger the diameter of soil particles, the more concentrated the distribution of soil pores, and the larger the size of soil pores. Comparing the soil particle movement states corresponding to different soil particle diameters, the soil particle movement corresponding to soil particle diameters of 4 and 5 mm has tended to stabilize, while a small number of soil particles corresponding to soil particle diameter of 6 mm are still in motion, indicating that as the soil particle diameter increases, the time required for soil cavity stability also increases. By comparing the size of soil cavity corresponding to different soil particle diameters, the maximum height and width of soil cavity corresponding to soil particle diameters of 6 mm are 14.81 and 41.18 mm, which are significantly larger than the corresponding values of other soil particle diameters. The maximum width of the soil cavity corresponding to the diameter of 4 mm is only 21.93 mm; the maximum width corresponding to the diameter of 6 mm is nearly twice that and the cavity sizes of soil particles with a diameter of 4 and 5 mm are relatively close.

**Figure 13.**
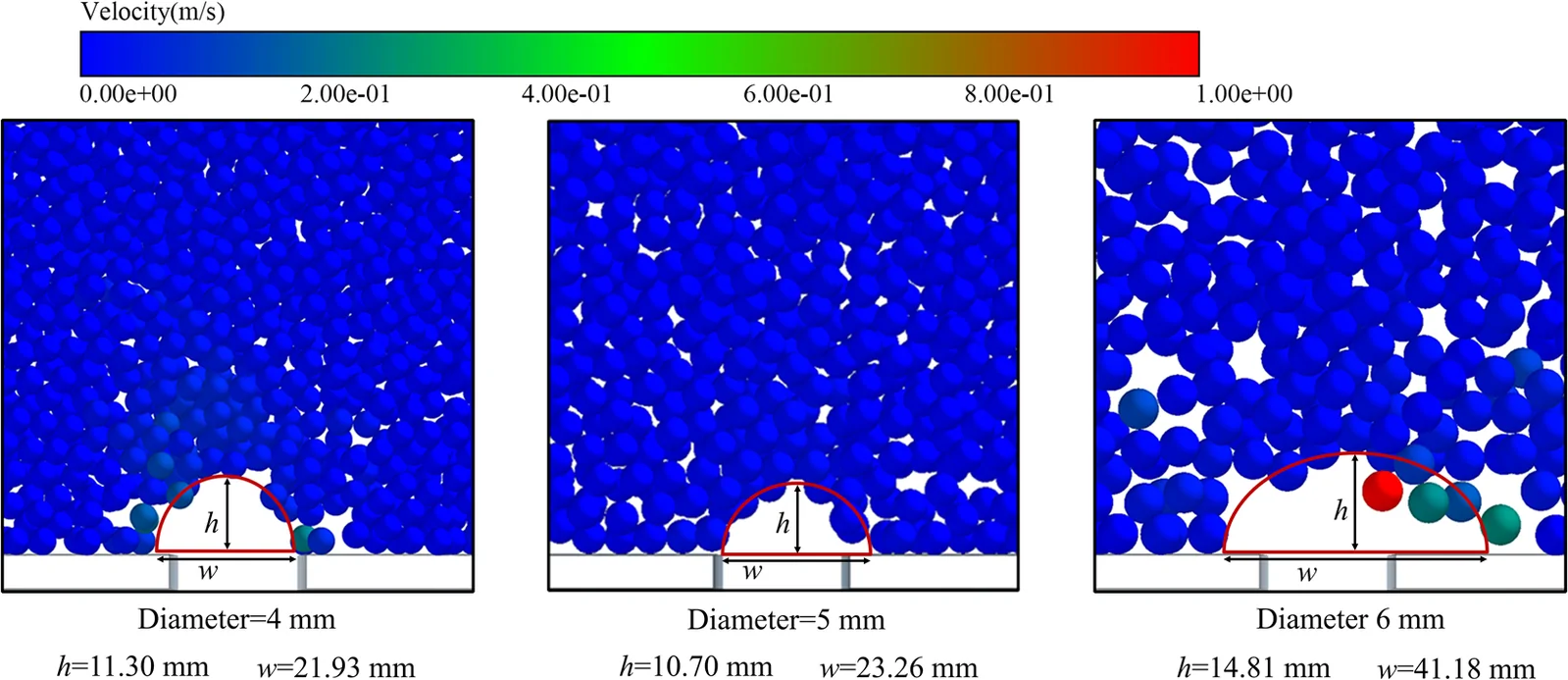
Soil cavity under different particle diameters.

#### 3.2.2. Different particle diameters correspond to leakage velocity and the highest dangerous range comparison

To explore the influence of soil particle diameter on gas leakage diffusion, leakage velocity and HDR corresponding to different soil particle diameters are shown in figure 14. Figure 14a shows the changes in gas leakage velocity corresponding to different soil particle diameters. The leakage velocity curve indicates that the stable gas leakage velocities corresponding to different soil particle diameters are very close. Comparing the leakage velocities corresponding to different particle diameters at the same time, as the diameter increases, the leakage velocities are 0.14576 m s^−1^ in sequence, 0.14609 and 0.14631 m s^−1^. The soil cavity size corresponding to the soil particle diameter of 6 mm is the largest, and its gas leakage velocity is also the largest. The size of the soil pore may affect the gas leakage velocity. Figure 14b shows the methane concentration distribution at monitoring line 1 corresponding to different soil particle diameters after pipeline leakage occurred for 0.1 s. The horizontal orange line in the figure corresponds to the lower explosion limit concentration of methane, and there are significant differences in methane concentration curves for different soil particle diameters. Within the range closer to the leakage port, the methane concentration value corresponding to small-diameter soil particles is significantly higher than that of large-diameter soil particles. When the distance from the leakage port is farther, the relationship between concentration values reverses and the methane concentration of small diameter soil particles is smaller than that of large diameter soil particles, indicating that when the diameter of soil particles is small, methane is more concentrated near the leakage port. Comparing the HDR corresponding to different soil particle diameters, a larger soil particle diameter corresponds to a larger HDR and a higher potential explosion risk caused by leakage.

**Figure 14.**
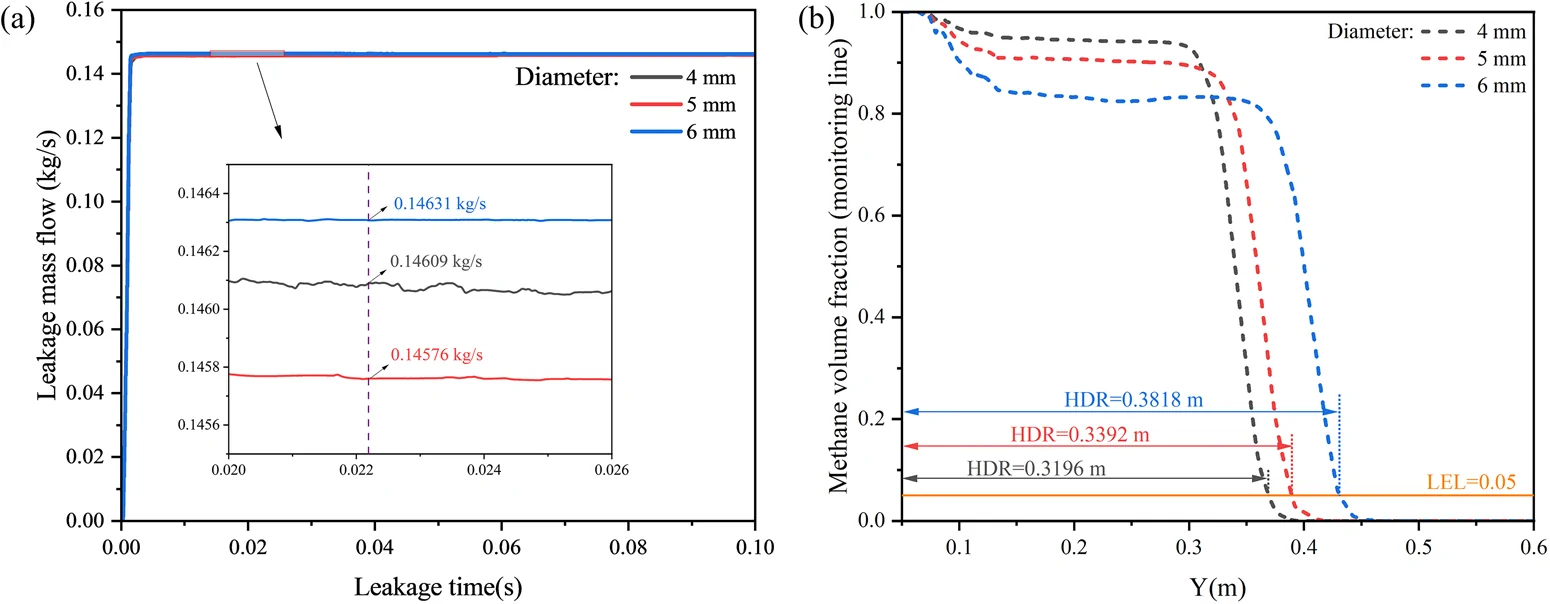
Leakage velocity and HDR corresponding to different particle diameters.

### 3.3. The influence of soil porosity on leakage process

#### 3.3.1. Different soil porosities correspond to soil cavity comparison

Figure 15 shows the size of soil cavity corresponding to different soil porosities after pipeline leakage occurred for 0.1 s. As shown in the figure, comparing the soil particle motion states corresponding to different soil porosities, the soil particle motion corresponding to soil porosities of 0.4 and 0.45 has tended to stabilize, while some soil particles corresponding to soil porosity of 0.5 are still in motion, that is, the soil cavity is not stable. This indicates that as the soil porosity increases, the time required for soil cavity stability also increases, which is consistent with the law shown by the change in soil particle diameter. By comparing the size of soil cavity corresponding to different soil porosities, the results showed that different soil porosities had a significant influence on the size of soil cavity. With the increase of soil porosity, the corresponding maximum height of soil cavity was 4.07, 10.70 and 15.09 mm, respectively, showing obvious regularity, indicating that with the increase of soil porosity, the size of soil cavity also increased. In connection with the analysis of soil particle diameter, the change in soil cavity size caused by soil particle diameter should be the result of the change of soil particle diameter changing soil internal pore structure, and soil particle diameter does not directly affect the size of soil cavity.

**Figure 15.**
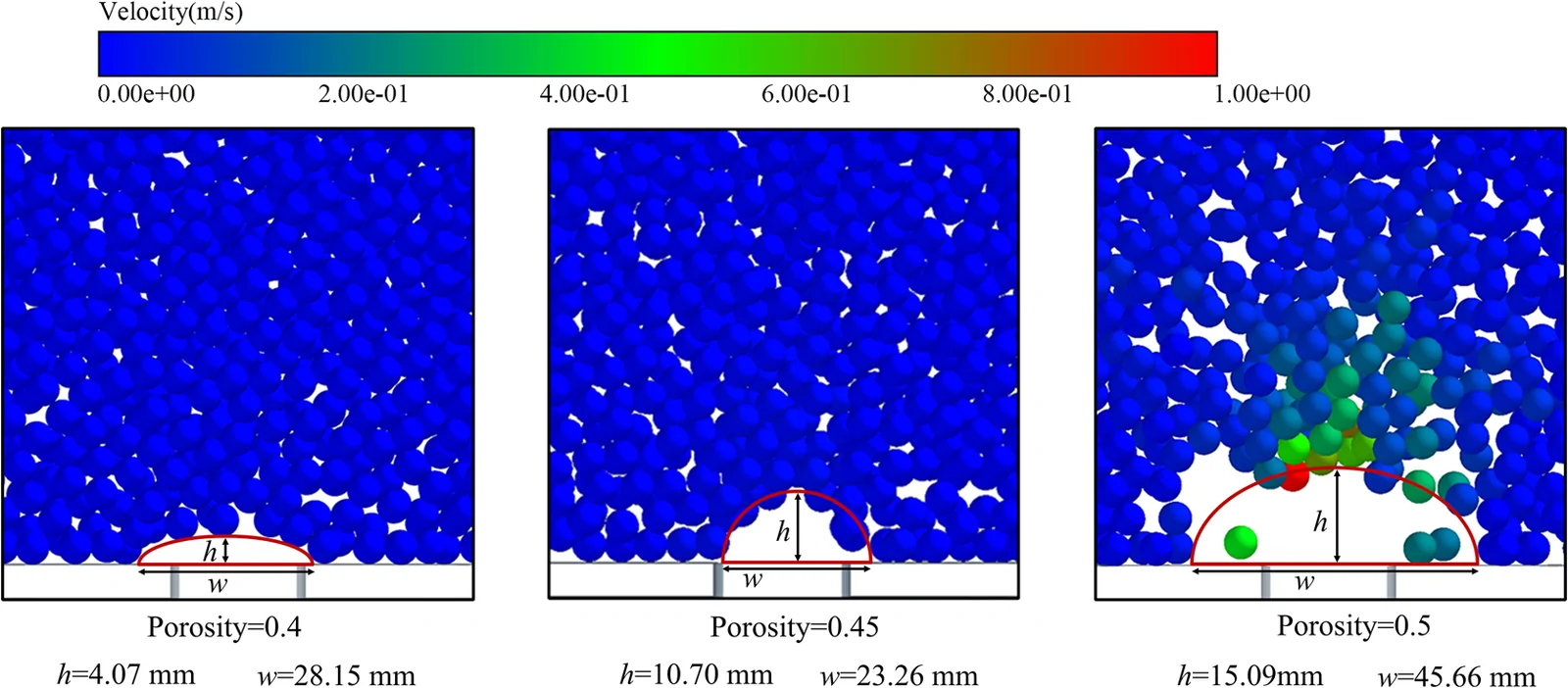
Soil cavity under different soil porosities.

#### 3.3.2. Different soil porosities corresponded to leakage velocity and highest dangerous range

To explore the influence of soil porosity on gas leakage diffusion, leakage velocity and HDR corresponding to different soil porosities are shown in figure 16. Figure 16a shows the variation of gas leakage velocity corresponding to different soil porosities. Comparing the leakage velocity curves at the same time, the curves indicate that although the gas leakage velocities corresponding to different soil porosities are very close, the leakage velocity is positively correlated with soil porosity. As the soil porosity increases, the gas leakage velocity also increases. Figure 16b shows the methane concentration distribution at monitoring line 1 corresponding to different soil porosities after pipeline leakage occurred for 0.1 s. The distribution law of methane concentration for different soil porosities and soil particle diameters is similar, and as the soil porosity increases, the corresponding HDR increases, and the potential explosion risk owing to leakage is higher.

**Figure 16.**
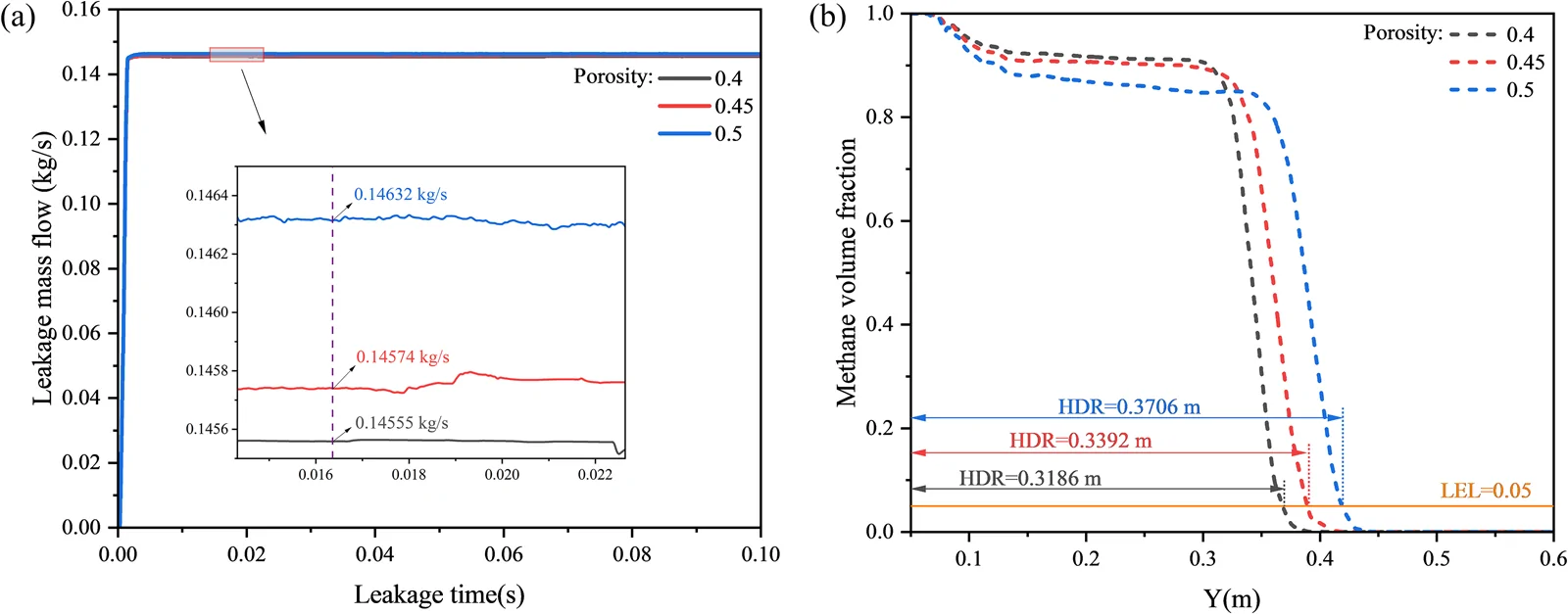
Leakage velocity and HDR corresponding to different soil porosities.

### 3.4. The influence of soil moisture content on leakage process

#### 3.4.1. Different soil moisture contents correspond to soil cavity comparison

Figure 17 shows the size of soil cavity corresponding to different soil moisture contents after pipeline leakage occurred for 0.1 s. By comparing the motion states of soil particles corresponding to different soil moisture contents, the motion of soil particles corresponding to each soil moisture content has been stabilized, indicating that soil moisture content has little influence on the formation time of soil cavity. By comparing the size of soil cavities corresponding to different soil moisture contents, the results showed that different soil moisture contents had a significant influence on the size of soil cavities. With the increase of soil moisture content, the corresponding maximum height of soil cavity was 8.61, 10.70 and 13.81 mm, respectively, showing obvious regularity, indicating that with the increase of soil moisture content, the size of soil cavity also increased.

**Figure 17.**
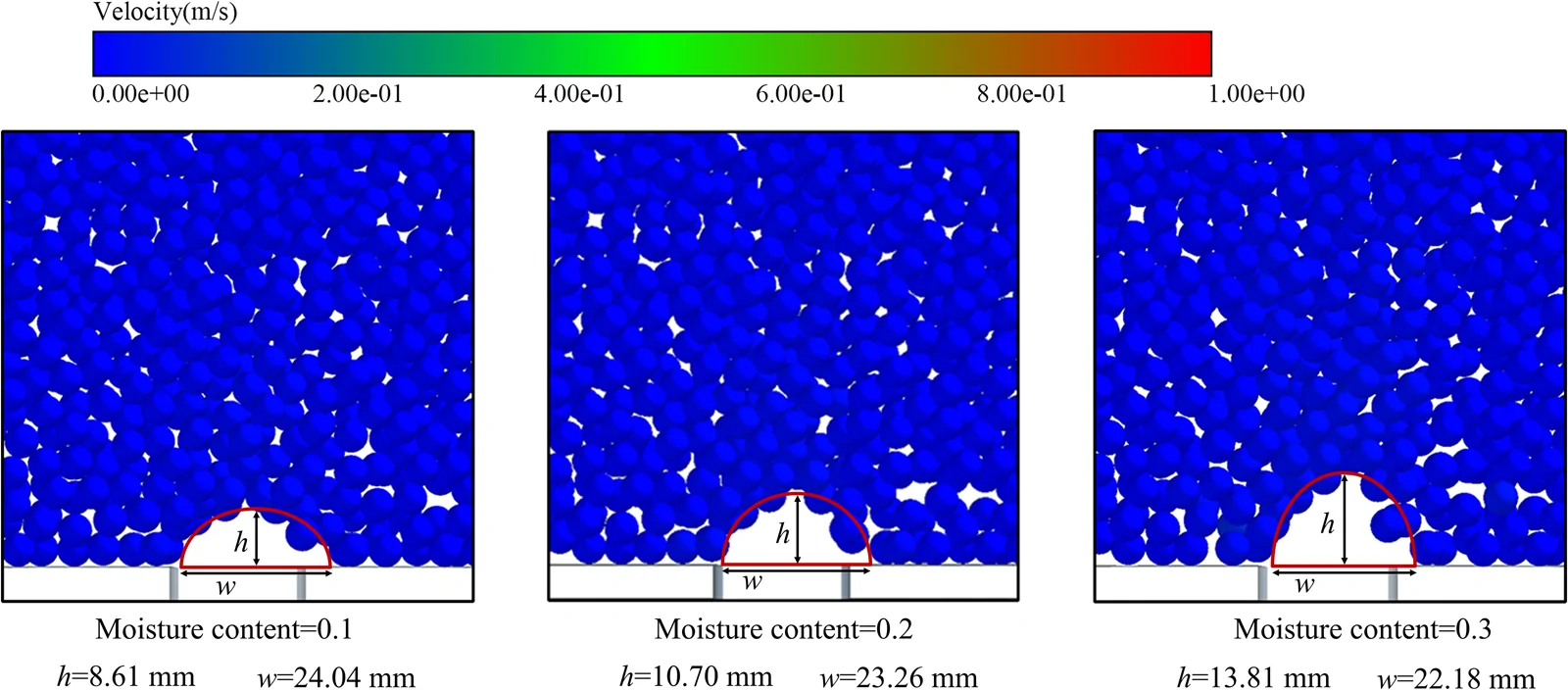
Soil cavity under different soil moisture contents.

#### 3.4.2. Different soil moisture contents corresponded to leakage velocity and highest dangerous range

To explore the influence of soil moisture content on gas leakage diffusion, leakage velocity and HDR corresponding to different soil moisture contents are shown in figure 18. Figure 18a shows the variation of gas leakage velocity corresponding to different soil moisture contents. The leakage velocity curve is consistent with the previous text. Although the gas leakage velocity corresponding to different soil moisture contents is very close, the leakage velocity is positively correlated with the soil moisture content. With the increase of soil moisture content, the gas leakage velocity also increases. Figure 18b shows the methane concentration distribution at monitoring line 1 corresponding to different soil moisture contents after pipeline leakage occurred for 0.1 s. The distribution of methane corresponding to different soil moisture contents is very similar. As the soil moisture content increases, the corresponding HDR values are 0.3387, 0.3392 and 0.3409 m, respectively. This indicates that as the soil moisture content increases, the corresponding HDR increases and the potential explosion risk caused by leakage is higher.

**Figure 18.**
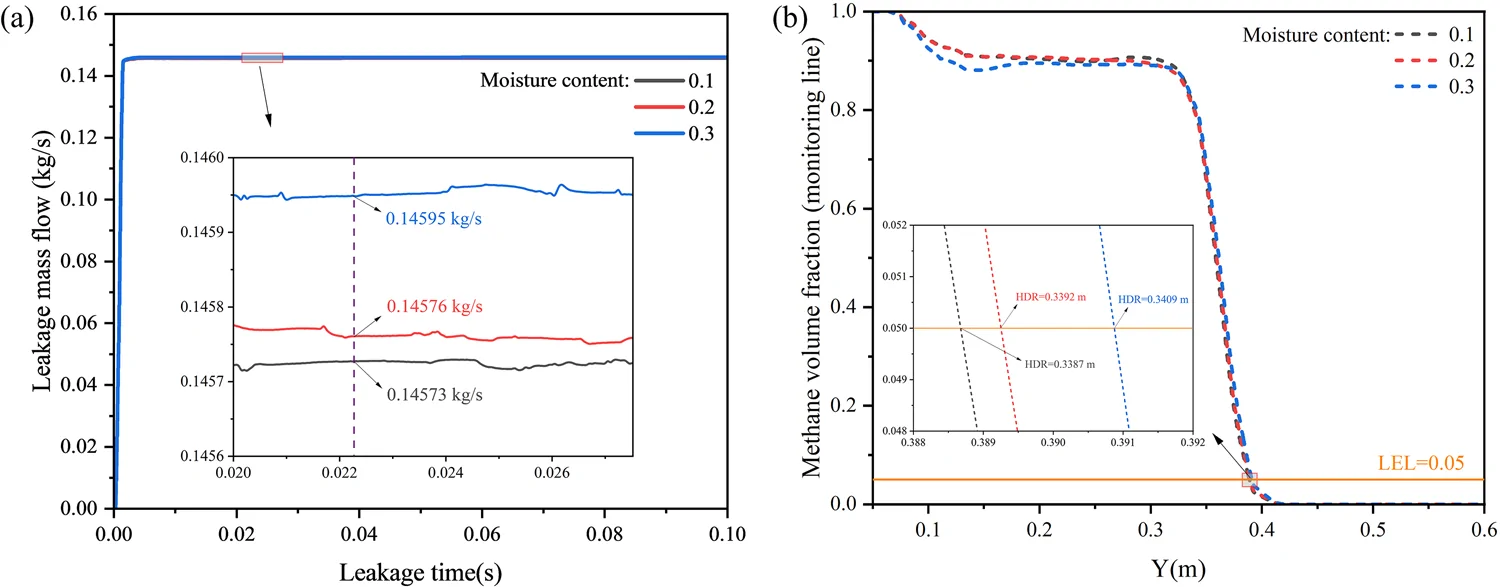
Leakage velocity and HDR corresponding to different soil moisture contents.

### 3.5. The influence of pipeline pressure on leakage process

#### 3.5.1. Different pipeline pressures correspond to soil cavity comparison

Figure 19 shows the size of soil cavity corresponding to different pipeline pressures after pipeline leakage occurred for 0.1 s. As shown in the figure, by comparing the motion states of soil particles corresponding to different pipeline pressures, the movement of soil particles corresponding to 0.2 MPa and 0.3 MPa tends to be stable, while some soil particles corresponding to 0.4 MPa are still in motion, that is, the soil cavity is not stable, indicating that the stabilization time of the soil cavity is also increasing with the increase of pipeline operating pressure. Comparing the sizes of soil cavity corresponding to different pressures, although the maximum height and width of soil cavity corresponding to a pressure of 0.3 MPa are very close to those corresponding to a pressure of 0.4 MPa, by observing the overall size of soil cavity, it can be found that the soil cavity corresponding to a pressure of 0.4 MPa is more complete and the contour of the soil cavity is closer to a semicircle. However, the soil cavity corresponding to a pressure of 0.3 MPa still has soil particles at the arc, indicating that the impact range of soil under a pressure of 0.4 MPa is wider.

**Figure 19.**
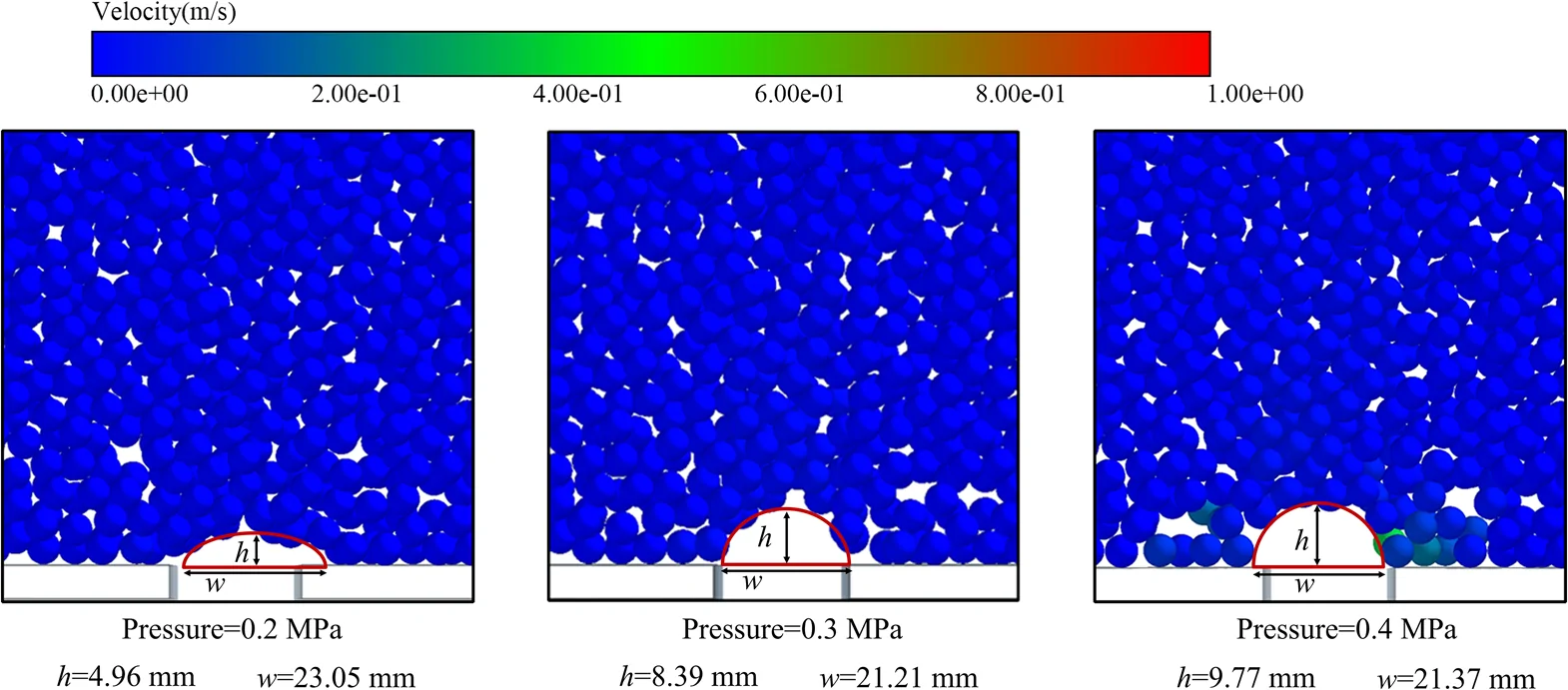
Soil cavity under different pipeline pressures.

#### 3.5.2. Different pipeline pressures corresponded to leakage velocity and highest dangerous range

To explore the influence of pipeline pressure on gas leakage diffusion, leakage velocity and HDR corresponding to different pipeline pressures are shown in figure 20. Figure 20a shows the variation of gas leakage velocity corresponding to different pipeline pressures. The leakage velocity curve indicates that there is a significant difference in gas leakage velocity corresponding to different pipeline pressures, and the leakage velocity is positively correlated with the pipeline pressure. As the pipeline pressure increases, the gas leakage velocity also increases. Figure 20b shows the methane concentration distribution at monitoring line 1 corresponding to different pipeline pressures after pipeline leakage occurred for 0.1 s. The distribution of methane corresponding to different pipeline pressures is very similar within the range of 0.2 m near the leakage port. As the diffusion distance increases, the methane concentration curve begins to show differences. With the increase in pipeline pressure, the corresponding HDR is 0.3222, 0.3392 and 0.3574 m, respectively, indicating that with the increase in pipeline pressure, the greater the corresponding HDR, the higher the potential explosion risk caused by leakage.

**Figure 20.**
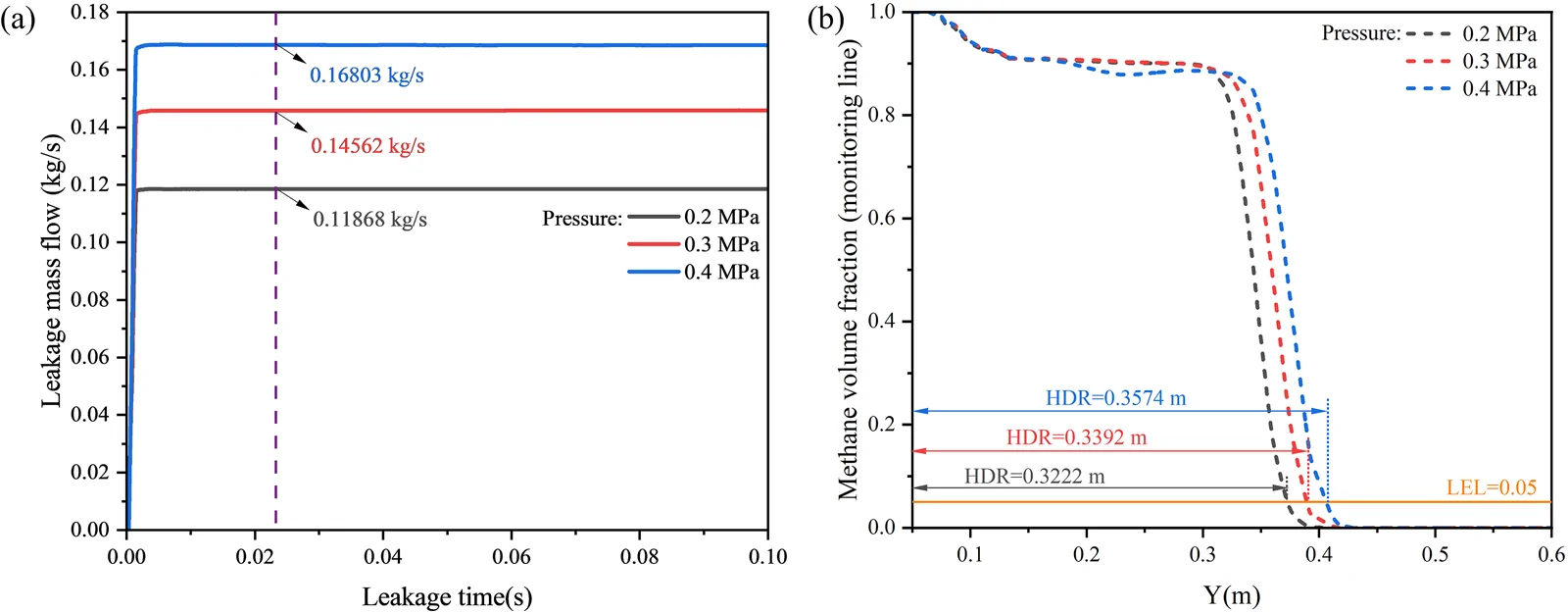
Leakage velocity and HDR corresponding to different pipeline pressures.

### 3.6. Analysis of the impact of various factors

The previous sections analysed the effects of soil particle diameter, porosity, moisture content and pipeline pressure on soil cavity size, gas leakage velocity and HDR. Figure 21 shows the statistics of the maximum height and width of soil cavity under various working conditions. The bar chart shows that the maximum width of soil cavity is generally greater than the maximum height, and the maximum width is greater than the diameter of the leakage port (20 mm). This is owing to the velocity direction of the leaked gas when it flows out of the pipeline leakage port being vertically upward. When the leaking gas is confronted with soil resistance, it begins to move horizontally around. Therefore, the leaked gas will lift the soil above the leakage port by a certain distance as a whole, resulting in the maximum width of the soil cavity formed being at least greater than the size of the leakage port. Comparing the maximum width of soil cavity under different working conditions, the maximum width corresponding to case 3 and case 5 is significantly higher than that of other working conditions. These two working conditions correspond to soil particle diameter of 6 mm and soil porosity of 0.5, respectively. According to the previous analysis, the increase in soil particle diameter changes the pore structure in the soil, making the distribution of soil pores more concentrated, while the increase in soil porosity directly increases the number of pores in the soil. This indicates that the pore structure and porosity of soil have a significant impact on the size of soil cavity.

**Figure 21.**
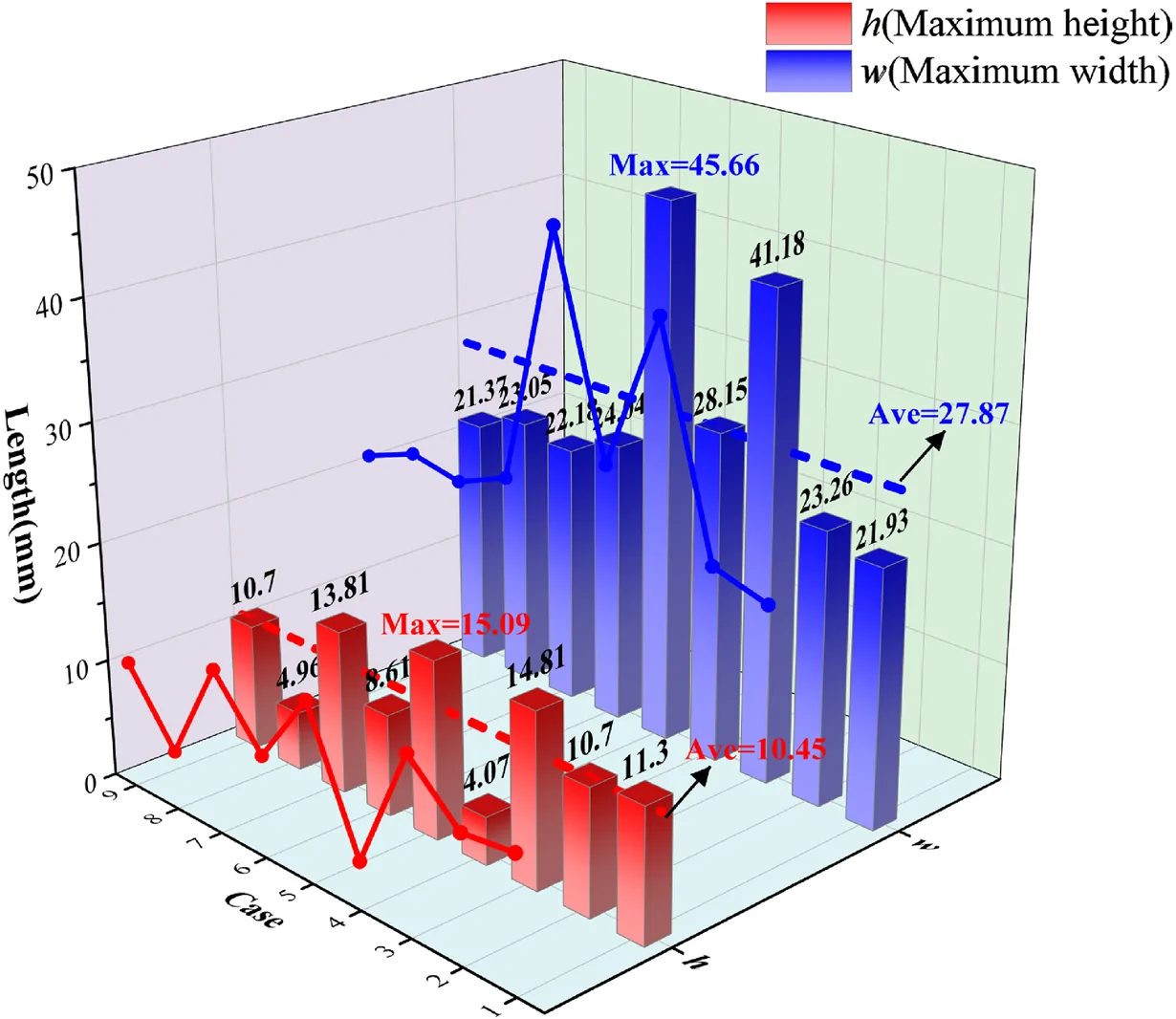
Maximum height and width of soil cavity under various working conditions.

To analyse the relationship between soil cavity size, gas leakage rate and HDR, sensitivity analysis was conducted on the maximum height and width of the soil cavity, and sensitivity coefficients *f* were introduced. The specific calculation process is shown in equation (2.23) [48]:

(2.23) $$f=\frac{\Delta i/i}{\Delta l/l},$$

where *l* and *i* are the size of soil cavity (*h* and*w*) and the gas leakage diffusion (gas leakage velocity and HDR) corresponding to the standard working condition; Δ*l*and Δ*i* are the corresponding changes when the working condition changes.

By equation (2.23), calculations show that the average sensitivity coefficients of the maximum height of soil cavity to gas leakage velocity and HDR are −1.39 and −0.87, respectively, the average sensitivity coefficients of the maximum width of soil cavity to gas leakage velocity and HDR are 5.71 and 18.78, respectively. The calculation results indicate that the maximum height of soil cavity formed after the gas pipeline leak is insensitive to gas leak rate and HDR, while the maximum width of soil cavity is positively correlated with gas leak rate and HDR. It can be considered that the larger the maximum width of soil cavity formed by pipeline leak impact, the higher the potential risk of leakage.

In the DEM model, particle positions are randomly generated and the random structure leads to differences in the distribution of local soil pores, which have a certain impact on the formation of soil voids and subsequent gas diffusion. Therefore, there is also a certain degree of uncertainty in the sensitivity coefficient. However, this uncertainty is caused by the random generation process of soil particles, and there are certain fluctuations in the impact under various working conditions. Based on this, the above sensitivity analysis focuses on exploring the relative influence trends of various factors, rather than absolute deterministic relationships. Although the random arrangement of particles may affect the local gas transport path, its impact is mainly concentrated on the local scale and will not change the overall trend. Therefore, the sensitivity results obtained can reflect the changing trend between the main influencing factors.

## 4. Conclusion

### 4.1. Main conclusions

This study conducted numerical simulation of a medium-pressure buried gas pipeline leakage based on CFD–DEM coupling. It explored the effects of soil particle diameter, porosity, moisture content and pipeline operating pressure on soil cavity and gas leakage diffusion, analysed the relationship between soil voids and gas leakage diffusion and obtained the following conclusions:

— after the leakage of medium-pressure buried gas pipeline, gas leaks into soil under the action of pressure difference inside and outside the pipeline, and the flow rate can reach more than 100 m per second in a very small range at the leakage port. Meanwhile, owing to the high-speed flow of leaking gas, negative pressure zone is formed around the leakage port. The soil structure above the leakage port is impacted by the leakage gas, forming a soil cavity is formed in a short time, which usually reaches a stable state within 0.1 s.
— The larger the soil particle diameter and porosity, the larger the size of soil cavity and the longer the time required for stability. It is worth noting that changes in soil particle diameter will affect the soil pore structure. The larger the particle diameter, the more concentrated the soil pore distribution and the larger the volume of a single pore, while the porosity will directly change the overall pore volume of the soil. Therefore, the interaction between soil particle diameter and soil porosity shows that the main factors affecting the formation of soil cavities are soil pore structure and pore volume.
— The leakage velocity is significantly positively correlated with the pipeline pressure, while changes in soil particle diameter, porosity and moisture content have little effect on the gas leakage velocity. HDR is positively correlated with all variables, with soil moisture having a relatively small impact on it.
— The influence of soil cavity size on gas leakage and diffusion was discussed and sensitivity analysis was conducted on both. The average sensitivity coefficient of the maximum height of soil cavity to gas leakage and diffusion is relatively low, while the average sensitivity coefficients of the maximum width of soil cavity to gas leakage velocity and HDR are 5.71 and 18.78, respectively. The greater the maximum width of soil cavities, the greater the gas leakage velocity and HDR. The results indicate that it is more appropriate to use the maximum width of soil cavity as the evaluation criterion when evaluating the size of soil cavity caused by medium-pressure gas pipeline leakage.
— The study is limited to medium-pressure buried gas pipeline and the soil structure deformation behaviour after leakage of high-pressure buried gas pipeline is significantly different from that of medium-pressure buried gas pipeline. The leakage situation of high-pressure buried gas pipeline needs further analysis.

### 4.2. Future outlook

The research results of this paper have certain guiding significance for the engineering design, operation monitoring and accident emergency of buried gas pipelines. The research shows that the soil pore structure and porosity are the main factors affecting the formation of soil cavities and gas diffusion after leakage. Therefore, in the process of pipeline construction and back-filling, the soil particle gradation and compaction density should be reasonably selected to reduce the risk of collapse and gas diffusion caused by excessive cavities after leakage. However, when considering the effect of moisture content on particle movement, this paper quantitatively characterizes different moisture contents by changing the friction coefficient between particles, ignoring the effects of capillary pressure, permeability evolution and water migration, making this model suitable for leakage in low- to medium-moisture-content soil.

In future research, the soil moisture model can be further improved, and a gas-liquid-solid coupling model considering the liquid bridge effect can be established by combining the multiphase flow theory and unsaturated soil mechanics model, so as to improve the applicability of the model in the real environment. Combined with the visualization experiment platform, the formation process of soil cavities, particle movement characteristics and gas leakage diffusion law of buried gas pipeline leakage can be verified. Further research can establish the quantitative relationship between the size of soil cavities and pipeline operation parameters, soil characteristics and other factors, establish the prediction model of cavity formation and gas diffusion range and provide a theoretical basis for real-time early warning and risk assessment of buried gas pipeline leakage accidents.

## Data Availability

Datasets and additional materials supporting this article are published on OSF [49].
